# Computational saturation mutagenesis to predict structural consequences of systematic mutations in the beta subunit of RNA polymerase in *Mycobacterium leprae*

**DOI:** 10.1016/j.csbj.2020.01.002

**Published:** 2020-01-17

**Authors:** Sundeep Chaitanya Vedithi, Carlos H.M. Rodrigues, Stephanie Portelli, Marcin J. Skwark, Madhusmita Das, David B. Ascher, Tom L. Blundell, Sony Malhotra

**Affiliations:** a Department of Biochemistry, University of Cambridge, Tennis Court Rd., CB2 1GA, UK; b Department of Biochemistry and Molecular Biology, Bio21 Institute, University of Melbourne, Parkville, VIC 3052, Australia; c Structural Biology and Bioinformatics, Baker Heart and Diabetes Institute, Melbourne, VIC 3004, Australia; d Molecular Biology Laboratory, Schieffelin Institute of Heath-Research and Leprosy Center, Karigiri, Vellore, Tamil Nadu 632106, India

**Keywords:** Mutation Coolspots, Mycobacterium leprae, In-silico Saturation Mutagenesis, Thermodynamic stability, Rifampin, RNA Polymerase

## Abstract

Rifampin resistance in leprosy may remain undetected due to the lack of rapid and effective diagnostic tools. A quick and reliable method is essential to determine the impacts of emerging detrimental mutations in the drug targets. The functional consequences of missense mutations in the β-subunit of RNA polymerase (RNAP) in *Mycobacterium leprae* (*M. leprae*) contribute to phenotypic resistance to rifampin in leprosy. Here, we report *in-silico* saturation mutagenesis of all residues in the β-subunit of RNAP to all other 19 amino acid types (generating 21,394 mutations for 1126 residues) and predict their impacts on overall thermodynamic stability, on interactions at subunit interfaces, and on β-subunit-RNA and rifampin affinities (only for the rifampin binding site) using state-of-the-art structure, sequence and normal mode analysis-based methods. Mutations in the conserved residues that line the active-site cleft show largely destabilizing effects, resulting in increased relative solvent accessibility and a concomitant decrease in residue-depth (the extent to which a residue is buried in the protein structure space) of the mutant residues. The mutations at residue positions S437, G459, H451, P489, K884 and H1035 are identified as extremely detrimental as they induce highly destabilizing effects on the overall protein stability, and nucleic acid and rifampin affinities. Destabilizing effects were predicted for all the clinically/experimentally identified rifampin-resistant mutations in *M. leprae* indicating that this model can be used as a surveillance tool to monitor emerging detrimental mutations that destabilise RNAP-rifampin interactions and confer rifampin resistance in leprosy.

**Author summary:**

The emergence of primary and secondary drug resistance to rifampin in leprosy is a growing concern and poses a threat to the leprosy control and elimination measures globally. In the absence of an effective *in-vitro* system to detect and monitor phenotypic resistance to rifampin in leprosy, diagnosis mainly relies on the presence of mutations in drug resistance determining regions of the *rpoB* gene that encodes the β-subunit of RNAP in *M. leprae.* Few labs in the world perform mouse food pad propagation of *M. leprae* in the presence of drugs (rifampin) to determine growth patterns and confirm resistance, however the duration of these methods lasts from 8 to 12 months making them impractical for diagnosis. Understanding molecular mechanisms of drug resistance is vital to associating mutations to clinically detected drug resistance in leprosy. Here we propose an *in-silico* saturation mutagenesis approach to comprehensively elucidate the structural implications of any mutations that exist or that can arise in the β-subunit of RNAP in *M. leprae.* Most of the predicted mutations may not occur in *M. leprae* due to fitness costs but the information thus generated by this approach help decipher the impacts of mutations across the structure and conversely enable identification of stable regions in the protein that are least impacted by mutations (mutation coolspots) which can be a potential choice for small molecule binding and structure guided drug discovery.

## Graphical abstract

**Figure:**
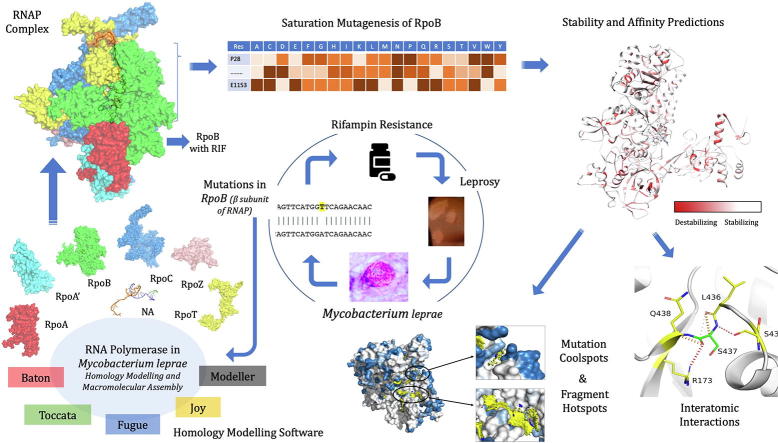

## 1 Introduction

Nonsynonymous mutations in genes that encode drug targets in mycobacteria can induce structural and consequent functional changes leading to antimicrobial resistance, the burden of which is rapidly increasing and is a global health concern. Diagnosis of ~600,000 new cases of rifampin-resistant tuberculosis in 2018 suggest that it poses a risk for the concomitant increase in undiagnosed rifampin-resistant leprosy, worldwide [1]. *Mycobacterium leprae (M. leprae),* the causative bacilli for leprosy, is phylogenetically closest to *Mycobacterium tuberculosis* [2] and developed resistance to rifampin before the introduction of World Health Organization (WHO) recommended multi-drug therapy (MDT) in the year 1984. Despite the long duration of chemotherapy with MDT (six months in paucibacillary to 12 months in multibacillary disease), rifampin-resistant case numbers are less and represent only 3-5% of total clinically diagnosed relapsed leprosy cases as reported by WHO in 2017 [3]. One of the possible reasons for the low numbers of drug-resistant leprosy cases globally is the lack of quick, effective and reliable *in-vitro* diagnostic test for confirming phenotypic resistance. Current methods rely on identifying mutations in the rifampin resistance determining region (RRDR) of the *rpoB* gene through gene sequencing and/or by testing growth patterns of *M. leprae* in response to individual drugs in the MDT in an *in-vivo* mouse footpad model; however, the later technique is both time and labour intensive.

While mutations within the β-subunit of RNAP contribute to clinical resistance to rifampin, the associated structural changes can complicate the transcription process in bacteria by modulating various physiological processes [4], the knowledge of which is essential for novel drug discovery or alternative therapies to treat rifampin resistant strains of *M. leprae*. In the absence of an artificial culture system to propagate and study mechanisms of resistance, it is exceptionally challenging to define an experimental phenotype for rifampin resistance in leprosy. *M. smegmatis* as a surrogate host with cloned *M. leprae rpoB* gene has proved a dependable model to study phenotypic effects; however, this technique is limited to biosafety level-2 laboratories that have facilities for gene cloning and sequencing, and cannot be translated to a regular diagnostic setting in leprosy endemic countries [5]. A plausible association between mutations in drug targets and phenotypic resistance outcomes could be established if minimum inhibitory concentrations (MICs) of the drugs are known for the mutant strains. While MICs can be estimated in cultivable species like *M. tuberculosis* and *M. smegmatis*, obtaining growth information from *in vivo* propagation for a slow growing and obligate pathogen like *M. leprae* is challenging and needs time and resources. Alternatively, *in-silico* methods that predict structural implications of mutations can be useful in understanding mechanisms of resistance and help prioritise mutations that require experimental validation in leprosy, owing to the absence of a tool for quantitative estimation of phenotypic resistance [6].

Mutations contribute to disruption of protein–ligand and protein-nucleic acid interactions resulting in drug resistance in mycobacterial diseases [7], [8]. Changes in affinity between the drug target protein and the ligand can result from both orthosteric and allosteric mechanisms leading to various resistance phenotypes [4]. The β-subunit of RNAP in *M. leprae* is encoded by the *rpoB* gene (ML1891) whose product is 1178 amino acids in length. The RRDR is located between the residue positions 410 and 480. Approximately 40 mutations have been reported in the *rpoB* gene of *M. leprae* that induce clinical resistance to rifampin in leprosy [9], [10], [11]; however, in tuberculosis, nearly 100 mutations have been reported in the same gene that shares 96% gene sequence identity with that of *M. leprae* [12]*.* As the burden of rifampin resistance is very high in *M. tuberculosis* with known and new mutations being reported from different studies [13], [14], [15], [16], [17], it is important to monitor the emergence of new rifampin-resistant mutations in *M. leprae*. A comprehensive understanding of the effect of any mutation on the structure of RNAP is vital in the context of monitoring emerging rifampin resistance and its implications on controlling global leprosy incidence.

In order to decipher the effect of systematic mutations on the stability of the protein structure, protein sub-unit interfaces, nucleic acid and ligand interactions, we performed *in-silico* saturation mutagenesis (mutating every residue to all the other 19 residues) and predicted the change in stability of the β-subunit and affinity between β-subunit andrest of the subunits in the complex, β-subunit-rifampin and β-subunit-RNA interactions. Additionally, we also assessed the impacts of mutations on the secondary structures of the polypeptide chains, relative sidechain solvent accessibility, residue-depth and residue-occluded packing density [18]. Residue-level evolutionary conservation scores were determined and compared with the predicted destabilizing effects. Extremely detrimental mutations (that destabilize β-subunit of RNAP and affinity between β-subunit -rest of the subunits in the complex, β-subunit -rifampin and β-subunit-RNA interactions) were selected and analysed for changes in their interatomic interactions that might explain the reasons for the predicted destabilizing effects. To explore further, the vibrational entropy and enthalpy changes of the protein in flexible conformations, we employed an empirical force field-based method – FoldX [19], a course-grained normal mode analysis (NMA) based elastic network contact model – ENCoM [20] and a consensus predictor that integrates normal mode approaches with graph-based distance matrix in the mutating residue environment– DynaMut [21]. Finally, fragment hotspots [22] were mapped on the structures to provide information on potential druggable sites whose stability is predicted to be least likely affected by mutations (no mutations in these regions were identified in leprosy). We termed these sites as “Mutation coolspots” which can be explored for novel/alternative small molecule binding and structure-guided drug discovery to treat rifampin-resistant leprosy.

## 2 Materials & Methods

### 2.1 Design:

**Fig. 1:**
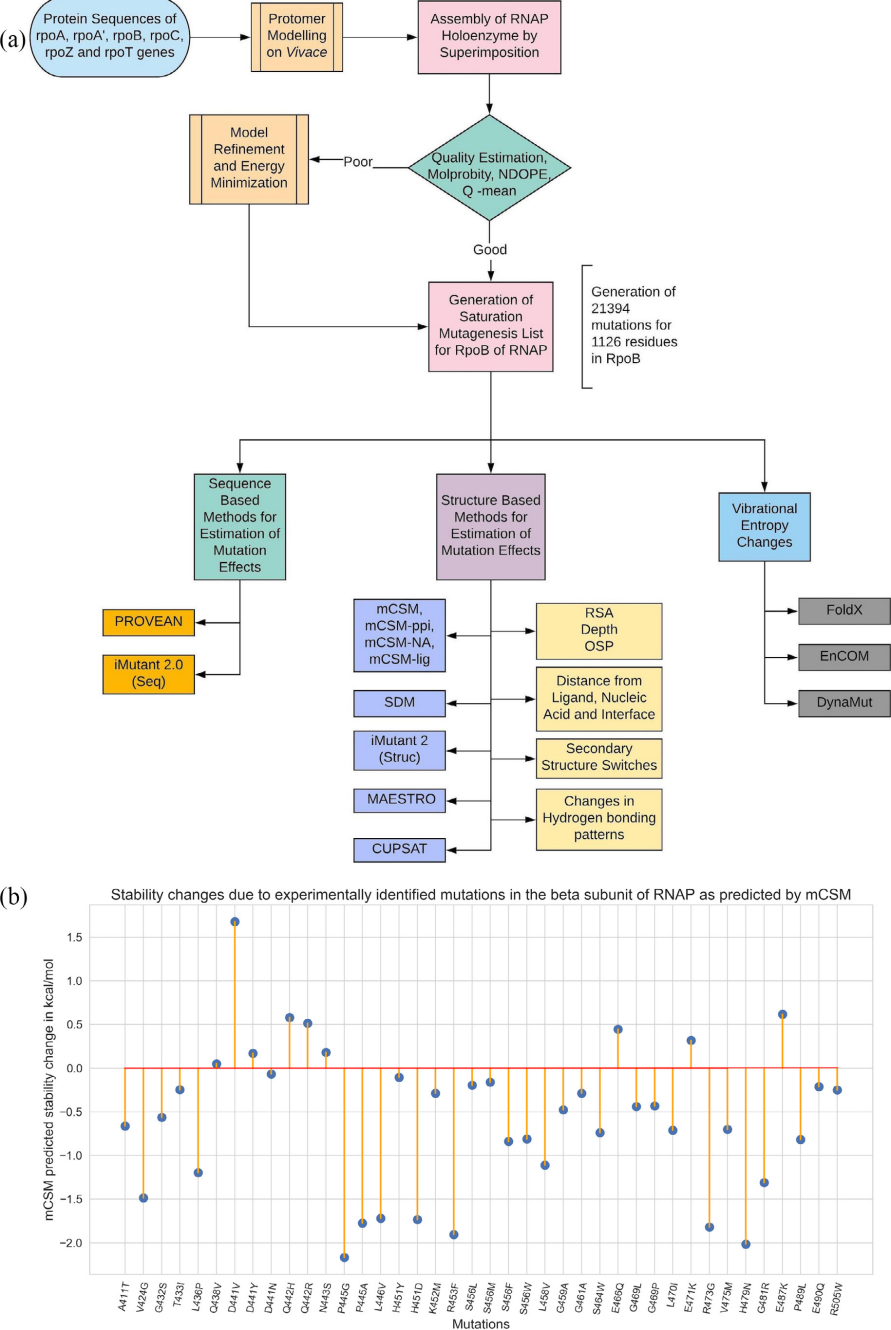
[A] Methodology and study design. [B] A lollipop plot with stability predictions for mutations reported in the literature and are known to confer rifampin resistance in Leprosy.

The key stages in the methodology involve comparative protein 3D modelling using known crystal structures of homologues as templates, quality assessment of the built models, generating mutation lists from the model and sequential submission of the lists and the model to stability change prediction servers for sequence, structure and vibrational entropic terms (Fig. 1A).

### 2.2 Comparative modelling, quality assessment and model refinement:

A model for RNAP holoenzyme of *M. leprae* was built using Modeller 9.21 [23] with templates from *M. tuberculosis* (PDB Id:5UH5 (96% identity, 3.74 Å resolution) containing RNAP, nucleic acid scaffold with DNA and three nucleotides of RNA complementary to the template DNA strand, and PDB Id: 5UHC (96% identity, 3.79 Å resolution) containing all the elements similar to 5UH5 and rifampin) as described earlier by us [4]. The quality of the generated model was assessed using Molprobity [24] and atomic clashes were removed by minimizing the energy of the model by 100 steps using Steepest Decent (step size = 0.02 Å) and by 10 steps (step size = 0.02 Å) using conjugate gradient methods. Energy minimizations were performed using UCSF Chimera [25]. The mutant models were generated using a script from Modeller 9.21 (mutate_model.py) and sidechains of the mutants were optimized using ANDANTE [26], a program that uses χ angle conservation criteria to optimize the sidechain rotamers. Multiple models were generated initially to test the variation in the modelling process. Structural similarity among the models was tested using root mean square deviation (RMSD) and TM-Align scores [27]. (Supplementary Figs. 4–6, and Supplementary Table 3).

### 2.3 Saturated Mutagenesis:

A systematic list of 21,394 mutations was generated for residues starting from P28 and ending at E1153 positions in the β-subunit (the modelled region). This list was programmatically submitted to a set of servers as stated in Table 1 below:

**Table 1:** List of servers used in the computational analysis:

Si No:	Name of web server	Function	Reference	Submission parameters
1	mCSM	Predict protein stability changes due to mutations.	[28]	Model PDB file, mutation and chain id.
2	SDM	Predict protein stability changes due to mutations.	[18]	Model PDB file, mutation and chain id.
3	mCSM-PPI	Predict stability of protein–protein interfaces due to mutations.	[28]	Model PDB file, mutation and chain id.
4	mCSM-NA2	Predict stability of protein-nucleic acid interactions due to mutations	[29]	Model PDB file, mutation, chain id and nucleic acid type.
5	mCSM-lig	Stability of protein–ligand interactions due to mutations	[30]	Model PDB file, mutation, chain id, three letter code of the ligand and ligand affinity in wild type structure in nM concentration.
6	FoldX4	Predict protein stability changes due to mutations.	[19]	Model PDB file, list of mutations and chain ids.
7	MAESTRO	Predict protein stability changes due to mutations.	[31]	Model PDB file, list of mutations and chain ids.
8	CUPSAT	Predict protein stability changes due to mutations.	[32]	Model PDB file, list of mutations and chain ids.
9	Imutant 2.0-Struc	Predict protein stability changes due to mutations.	[33]	Model PDB file, list of mutations and chain ids.
10	Imutant 2.0 -Seq	Predict protein stability changes due to mutations using sequence information.	[33]	RNAP sequence file in fasta format, list of mutations and chain ids.
11	PROVEAN	Predict protein stability changes due to mutations using sequence information.	[34]	RNAP sequence file in fasta format, list of mutations and chain ids.
12	CONSURF	To calculate evolutionary conservation score of each residue in the protein.	[35]	Model PDB file
13	ENCoM	Conformational Changes in protein due to mutations.	[20]	Model PDB file, list of mutations and chain ids.
14	DynaMut	Conformational Changes in protein due to mutations.	[21]	Model PDB file, list of mutations and chain ids.
15	Arpeggio	Map interatomic interactions between wildtype and mutant amino acids and the residue environment.	[36]	Model PDB file and the residue selection in standard format.
16	Intermezzo	Map interatomic interactions between wildtype and mutant amino acids and the residue environment.	Bernardo Ochoa Montano & Blundell TL unpublished	Model PDB file and the residue selection in standard format.
17	ANDANTE	Works along with Modeller to generate mutant models from wildtype model files.	[26]	Model PDB file, mutation and chain id
18	Fragment Hotspot Maps	Maps regions on the surface of the protein that has high propensity for small molecule binding.	[22]	Model PDB file.

### 2.4 Residue conservation:

Conservation scores for each residue in the wild-type model was estimated using CONSURF – a server that uses evolutionary patterns of amino acids/nucleic acids from the multiple sequence alignment and develops a probabilistic framework to calculate evolutionary rates for each residue in the sequence.

### 2.5 Effects of mutations on protein stability and interactions:

The effect of mutations on thermodynamic stability of the β-subunit of RNAP was analyzed using mCSM, SDM and FoldX4. For SDM, mutant models were generated using ANDANTE. The effect of mutations on RNA affinity is assessed using mCSM-NA2 on mutant models with nucleic acid scaffold. The holoenzyme complex of RNAP consists of five subunits and the effects of mutations on the protein–protein interfaces (between β and all the other sub-units in RNAP complex) were assessed using mCSM-ppi. Rifampin binds to the β-subunit of RNAP and we analyzed the effects of mutations on the protein–ligand affinity using mCSM-lig. Only residues that are within 10 Å of interatomic distance to rifampin were analyzed by mCSM-lig.

The stability changes were further compared with predictions from other sequence- (PROVEAN, I-Mutant 2.0 (Sequence) and structure-based (MAESTRO, CUPSAT, I-Mutant 2.0 (Structure)) computational tools in order to estimate the reliability of the predictions.

### 2.6 Changes in vibrational entropy and normal mode analysis:

In order to determine the effects of the mutations in flexible conformations of the protein, we used FoldX4, an empirical force field approach that calculates free energy changes between native and mutant forms of the protein, and an elastic network contact model (ENCoM), which is a coarse grain NMA method that considers the nature of the amino-acids and aids in calculating vibrational entropy changes upon mutations. We also used DynaMut, a consensus predictor of protein stability based on the vibrational entropy changes predicted by ENCoM and the stability changes predicted by graph-based signatures that are used in mCSM program.

### 2.7 Conformational changes:

Conformational changes and their impacts on biophysical properties of the proteins were estimated using SDM. The interatomic distances between each residue and the interface with other subunits in the RNAP holoenzyme, rifampin and nucleic acids in the structure were measured and included in the analysis. Secondary structure switches in mutants, changes in relative solvent accessibility, depth of the residue in Å and residue-occluded packing densities were determined for all the mutations.

### 2.8 Interatomic interactions:

After predicting protein stability changes and changes in RNAP-rifampin affinities, mutations at two positions vide H451 & P489 that highly destabilize rifampin binding and are experimentally identified in the rifampin resistant leprosy patients [9], [10] (present in the set of 40 experimentally identified mutations – Supplementary Table 2), were analyzed for the changes in interatomic interactions of the mutating residues using Arpeggio, a program that maps the types of interatomic interactions of wildtype and mutant residues with the residue environment based on atom type, interatomic distance and angle constraints. Additionally, four mutations at positions S437, G459, K884 & H1035 which are computationally predicted to highly destabilize RNAP-rifampin interactions were chosen and subjected to similar analysis. Intermezzo program (Bernardo Ochoa Montano & Blundell TL unpublished) was also used for interactive analysis of bonding patterns on Pymol sessions.

### 2.9 Fragment hotspot maps:

Fragment hotspot maps aid in locating specific sites on the surface of the protein that are topologically, chemically and entropically favorable for small molecule (fragment) binding. The atomic hotspots on the drug target are explored computationally using donor, acceptor and hydrophobic fragment probes, and introducing a depth criterion to assist in estimating the small molecule binding propensity. For ligand-binding proteins, the fragment hotspot maps aid in understanding the pharmacophore characteristics of the interacting regions. We mapped the hotspots on the β-subunit of RNAP and colored the surface with regions that are least impacted by any mutations (mutation coolspots).

## 3 Results

In total, 21,394 mutations were generated from 1126 residues in the β-subunit of RNAP (Supplementary Table 1). The list of experimentally identified mutations and their effects are separately shown in Supplementary Table 2.

### 3.1 Multivariate analysis of free energy change predictions by various computational tools for saturated mutations:

**Supplementary figure 1:**
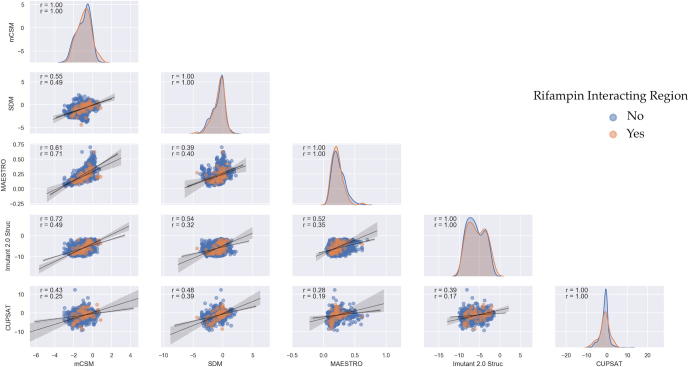
Pairplot depicting correlations between mCSM, SDM and other structural predictors of protein stability changes upon mutations in the b-subunit of RNAP. Each datapoint corresponds to maximum destabilizing effect noted at each residue position in the b subunit when systematically mutated to other 19 residues. The data points in orange correspond to predictions at rifampin interacting residues.

**Supplementary figure 2:**
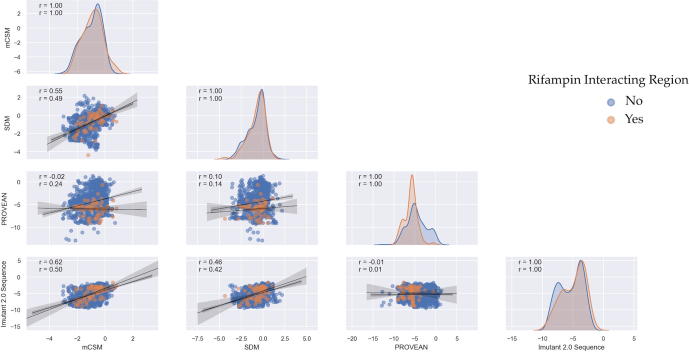
Pairplot depicting correlations between mCSM, SDM and other sequence-based predictors of protein stability changes upon mutations in the b-subunit of RNAP. Each data point corresponds to maximum destabilizing effect noted at each residue position in the b subunit when systematically mutated to other 19 residues. The data points in orange correspond to predictions at rifampin interacting residues.

**Supplementary figure 3:**
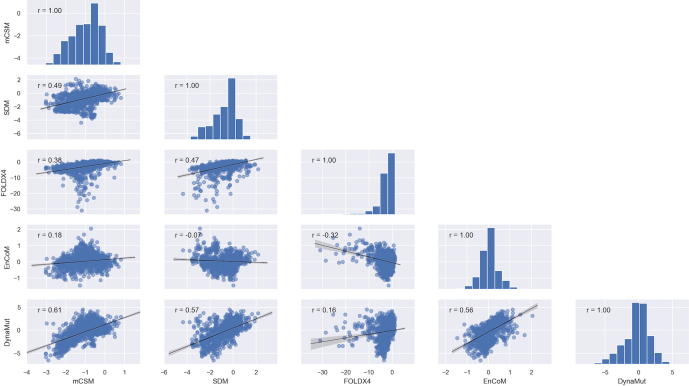
Pairplot depicting correlations between mCSM, SDM and other NMA-based predictors of protein stability changes upon mutations in the beta-subunit of RNAP. Each data point corresponds to average destabilizing effect noted at each residue position in the beta-subunit when systematically mutated to other 19 residues.

**Supplementary figure 4:**
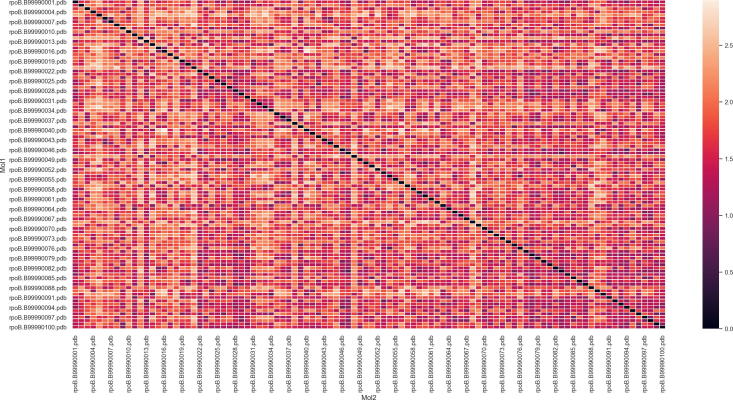
Heatmap showing variation in the RMSD among 100 models generated to validate the computational modelling of the beta-subunit of RNAP.

**Supplementary figure 5:**
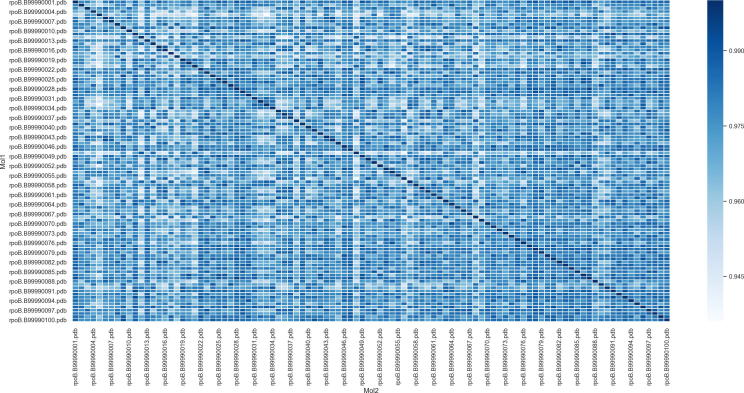
Heatmap showing variation in the TM-Align score among 100 models generated to validate the computational modelling of the beta-subunit of RNAP.

**Supplementary figure 6:**
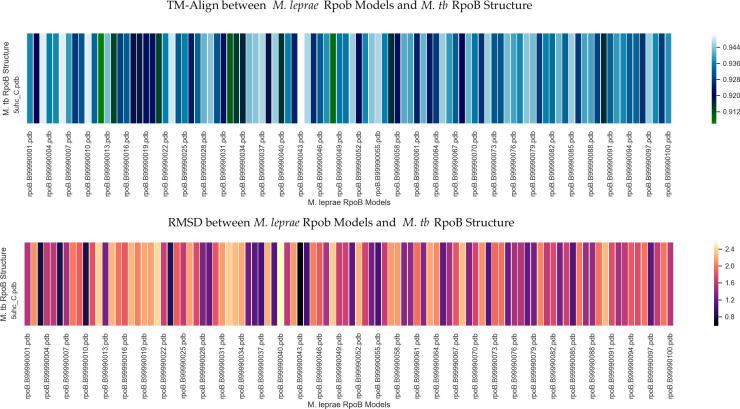
Heatmaps showing RMSD and TM-Align scores between <span data-format=*M*. *leprae* b-subunit of RNAP models and <span data-format=*M. tb* beta-subunit of RNAP structure (PDB Id: 5UHC).

Along with the in-house developed mCSM and SDM tools for prediction of protein stability changes upon saturated mutagenesis of the β-subunit of RNAP*,* a comparative analysis was performed with other sequence (PROVEAN, I-mutant 2.0 – Sequence), structure- (CUPSAT, I-mutant 2.0-structure, MAESTRO) and NMA-based tools (FOLDX, ENCOM, DynaMut). Average stability changes caused by all possible mutations at each residue position in the β-subunit of RNAP, as predicted by mCSM and SDM, were compared with other structure-based predictors (Supplementary Fig. 1) (rifampin-interacting residues are highlighted). Correlation of overall stability predictions performed by mCSM with each of the other tools indicated an “r” value of 0.55 with SDM, 0.61 with MAESTRO, 0.72 with Imutant 2.0 (Structure) and 0.43 with CUPSAT. Correlations between mCSM, SDM and other sequence and NMA based tools are shown in Supplementary Figs. 2 and 3. The rationale for performing these correlations is to understand how mCSM and SDM being structure-based predictors of stability changes, relate to sequence-based methods and vibrational entropy changes in normal mode perturbations.

### 3.2 Experimentally/Clinically identified mutations:

**Fig. 2:**
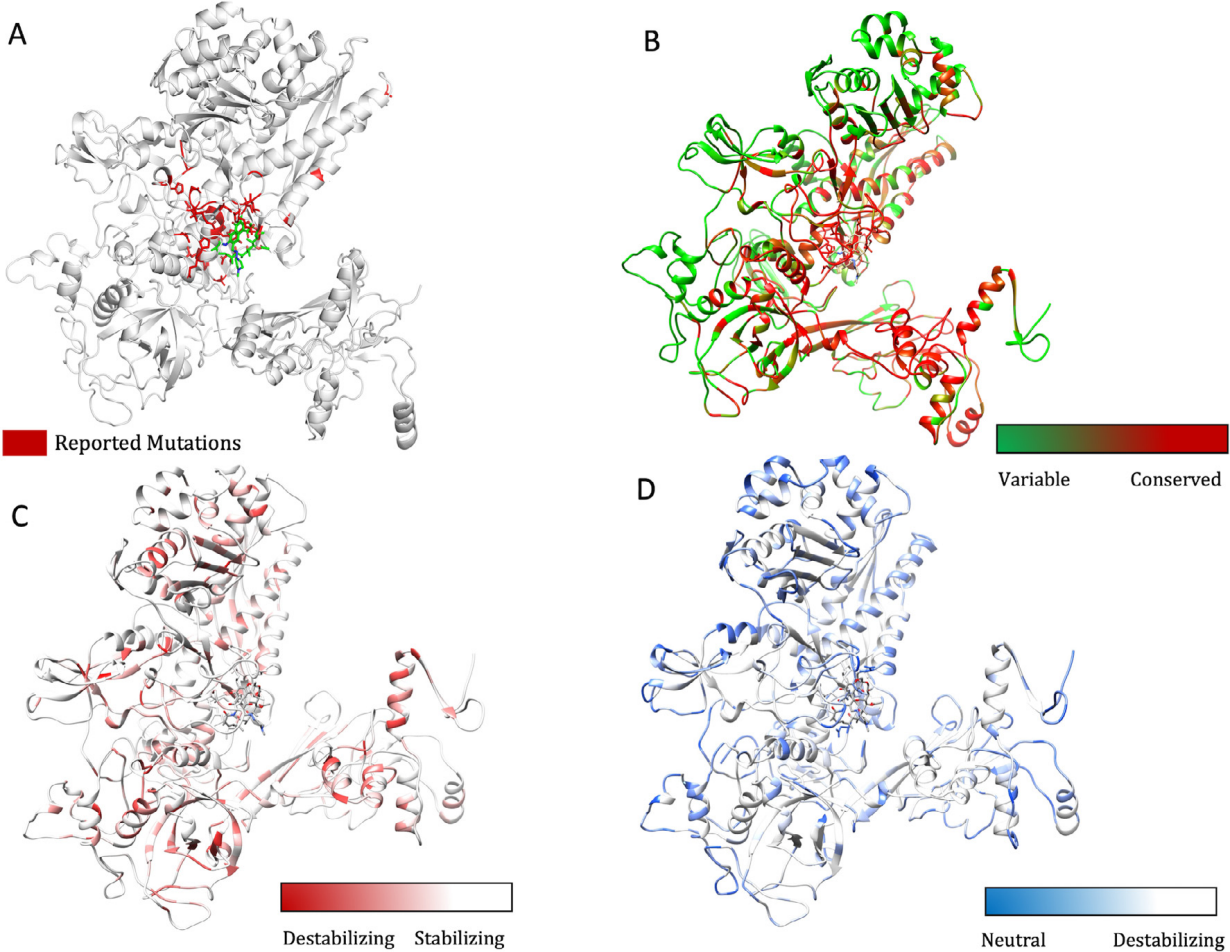
[A] The β-subunit of RNAP with residues where mutations were reported experimentally from patient samples in various studies (Supplementary Table 2) (highlighted in red). [B] Each residue in the β-subunit of RNAP that is colored by the conservations scores determined by CONSURF. The residues in green are variable (conservations scores greater than 1) and are usually surface exposed. The residues in red are conserved with conservation scores less than 1 and usually form the core of the protein. The rifampin binding site is highly conserved in *M. leprae.* [C] The maximum destabilizing effect (predicted by mCSM) on the protein stability for any mutation at each residue position, is mapped on the structure. Red are the regions that are largely destabilized by mutations while the white regions are relatively stable with mutations. [D] The converse of B where the regions, whose stability is least impacted by mutations, are coloured in blue and we called them “Mutation CoolSpots”. (For interpretation of the references to colour in this figure legend, the reader is referred to the web version of this article.)

We performed a systematic literature review to list all the mutations reported in the β-subunit of RNAP in *M. leprae*. We noted 40 mutations at 32 unique residue positions. The reference articles are listed in Supplementary Table 2. As depicted in Fig. 1B, 77.5% [19] of the experimentally/clinically identified mutations destabilize the β-subunit. Except for A411T and V424G mutations, all the other residues are present in close proximity to rifampin binding sites (Fig. 2A) and destabilize rifampin interactions (as predicted by mCSM-lig).

### 3.3 Residue conservation and protein stability:

The stability changes, predicted after saturation mutagenesis of each residue in the β-subunit, were compared with residue conservation scores. CONSURF scores of less than zero are attributed to conserved residues and scores of zero and above to variable residues (score 3 being highly variable). The average change in protein stability that was predicted by mCSM for mutations at each residue position ranged from 0.823 to −3.033 kcal/mol and that of SDM varied from 2.167 to −4.36 kcal/mol. Residues that line the active center cleft and interact with rifampin and the nucleic acid scaffold are highly conserved, while surface exposed residues have variable conservation scores (Fig. 2B). Rifampin-interacting residues between positions ~400–500 are highly conserved and 87.3% of the saturated mutations in this region destabilize the protein (Supplementary Table 1). The maximum destabilizing effect of mutations at each of these residues varied between −0.311 to −4.311 kcal/mol (mCSM). The maximum destabilizing mutation is defined as a mutation that induces a maximum decrease in Gibbs free energy (stability change) of the β-subunit of RNAP, RNAP-rifampin and RNAP-subunit interactions among all the 19 possible mutations at each residue position (when predicted by mCSM, SDM, mCSM-lig and mCSM-ppi software). The maximum destabilizing effect predicted by mCSM for all possible mutations at each residue was mapped on the structure to identify regions that are largely impacted by mutations (Fig. 2C). Conversely, the residues whose stability is least impacted by all possible mutations are colored in blue to identify “mutation coolspots” that are potentially areas of choice for targeting with small molecules in drug discovery (Fig. 2D).

**Fig. 3:**
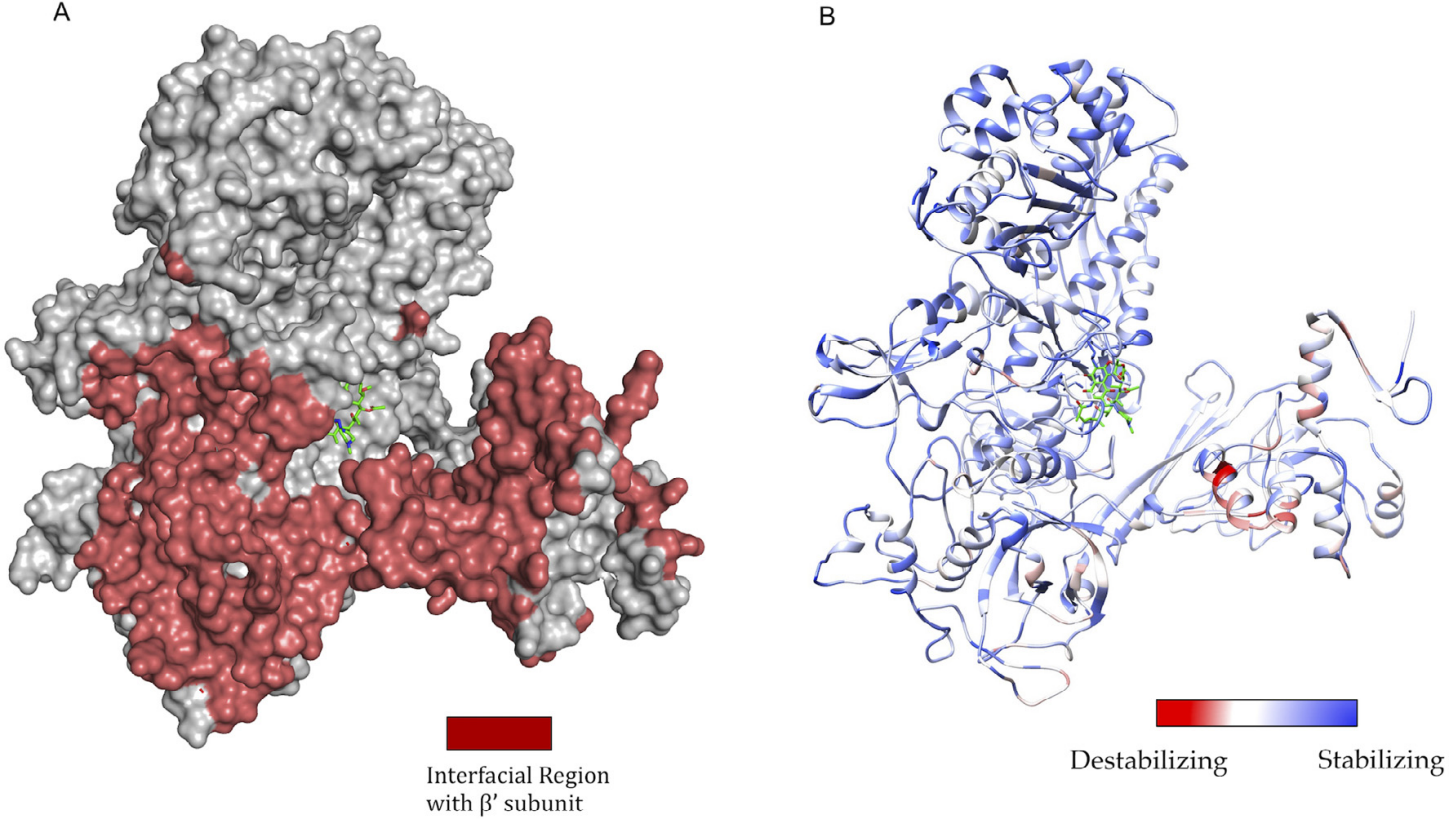
[A] The interfacial region of the β-subunit of RNAP highlighted in Maroon. [B]. The maximum destabilizing effect a mutation can induce on the interface stability, is predicted by mCSM-PPI and mapped on the structure. Red indicates regions that are highly destabilized by mutations (-5.108 Kcal/mol) while the blue indicates stable regions. (For interpretation of the references to colour in this figure legend, the reader is referred to the web version of this article.)

As part of the RNAP holoenzyme complex, the β-subunit interacts with other subunits and has large interfacial regions. The impact of mutations on the stability of these interfaces was measured using mCSM-PPI. It was noted that the maximum destabilizing effect by any mutation at a particular residue in the interface between β and β′ subunits has an affinity change that ranged from −0.021 to −5.108 kcal/mol (−5.108 kcal/mol was noted for mutation W1074R which is not reported experimentally in rifampin resistant leprosy cases). The interfacial region and the stability changes are mapped on the structure (Fig. 3A and B).

### 3.4 Relative sidechain solvent accessibility (RSA), residue-depth, residue-occluded packing density and protein stability:

**Fig. 4:**
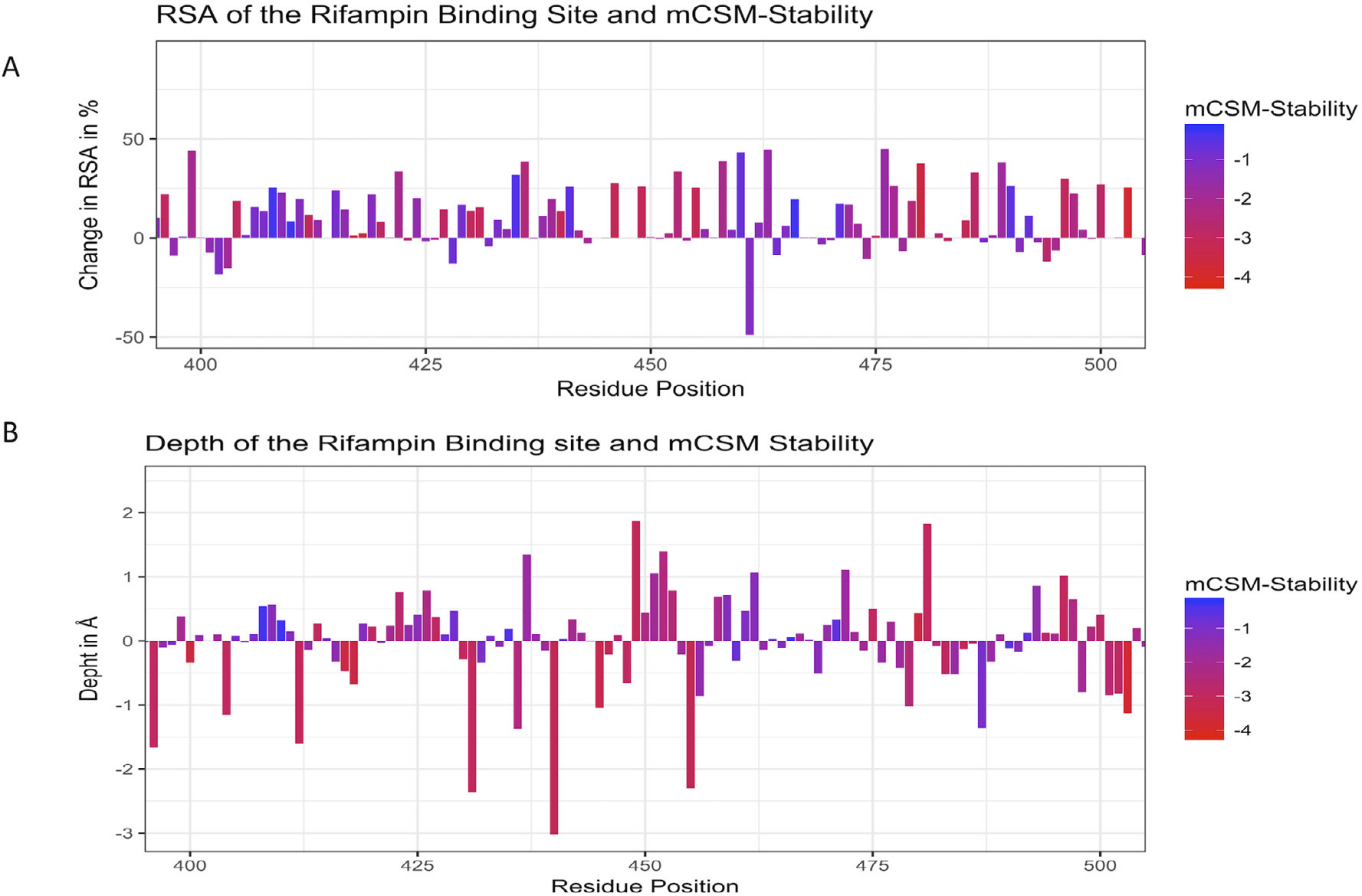
[A] Change in relative solvent accessibility for maximum destabilizing mutants in the rifampin binding pocket (mCSM). [B]. Change in depth of the highly destabilizing mutant residue in the rifampin binding pocket (mCSM).

The difference in relative solvent accessibility between wild type and the mutant residues for all the mutations were calculated using SDM. While analyzing the maximum destabilizing mutations among all the possible mutations at each residue position, it was noted that maximum destabilizing mutants at 751 residue positions (66.79%) showed increase in RSA. The maximum destabilizing mutants at rest of the 375 positions indicated a decrease in RSA. Among the 751 mutants with increase in RSA, 551 were hydrophobic and 121 substitutions within 551 were from polar/charged (wildtype) to hydrophobic residues (mutants). As mutant hydrophobic residues with increased solvent accessibility often destabilize the protein [38], the destabilizing effects of these mutations ranged from −1.021 to −4.311 kcal/mol. Additionally, these substitutions resulted in a decrease in residue-depth [18] (ranging from 0.01 Å to 1.83 Å), which is concomitant with the increase in solvent accessibility. These changes in RSA and depth at the rifampin-binding site are depicted in Fig. 4A and B.

**Fig. 5:**
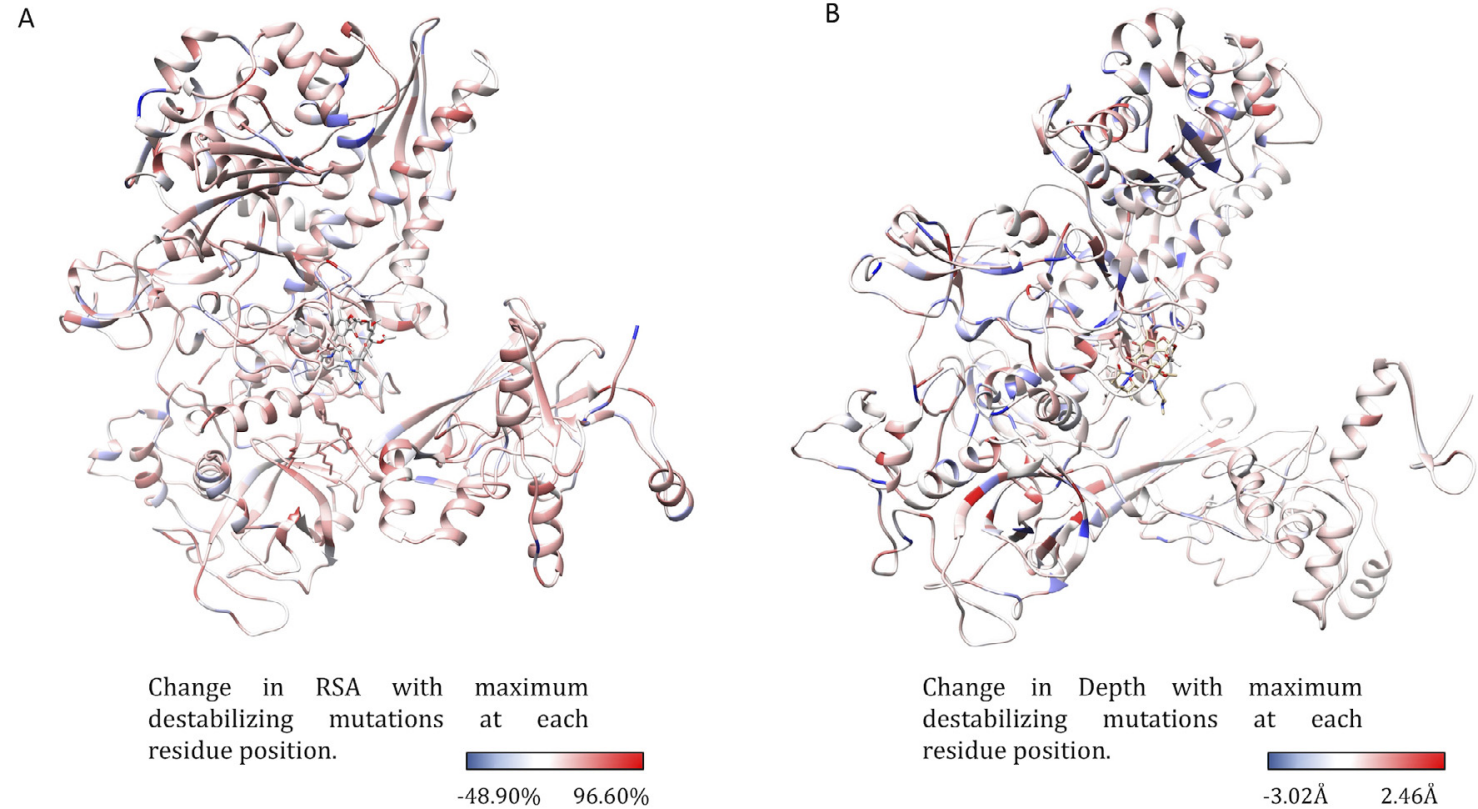
[A] The change in relative side chain solvent accessibility with mutations was mapped on to the structure. Blue indicates a decrease in RSA while red indicates an increase. [B] The changes in depth with highly destabilizing mutations at each residue position was mapped on the structure. (For interpretation of the references to colour in this figure legend, the reader is referred to the web version of this article.)

From the maximum destabilizing mutations at all the 1126 positions, mutations at 586 (52.04%) residue positions resulted in increase in residue-depth that ranged from 0.01 to 2.46 Å. Mutants were generated using ANDANTE which places the side chains without any steric clashes and the mutant models were subjected to energy minimization. Hence the change in residue-depth is attributed to the buriedness of the residue and not just the natural change from a larger to a smaller amino acid. The decrease in residue-depth in the remaining 540 (47.95%) residues ranged from 0.1 to 3.02 Å. Similarly, the residue-occluded packing density [18] increased at 539 residue positions (47.86%). These changes in RSA and residue-depth are mapped as attributes on to the structure of the β-subunit of RNAP and it was noted that most of the residues that line the active center cleft have increase in RSA upon mutations. Decrease in residue-depth was noted in residues at the rifampin-binding pocket and at the subunit interfaces (Fig. 5A and B).

### 3.5 Substitutions to aspartate predominate mutations that destabilize the β-subunit-RNA affinity in RNAP:

The effects of mutations on β-subunit-RNA affinity was estimated using mCSM-NA2. Substitutions to aspartate residues were most common among mutations that highly destabilize β-subunit-RNA interactions in RNAP. The mutant aspartate residues form π-π interactions with the nucleotides in RNA either by stacking or by nucleotide-edge T-shaped and amino-edge T-shaped interactions. Aspartate being an acyclic π-containing amino acid, readily forms nucleotide (edge) amino (edge) or nucleotide (face) and amino-acid (edge) interactions [39]. This ability of acyclic amino acids like arginine, glutamic acid and aspartic acid to form a variety of charged-π interactions with nucleotides in mutants may impact the orientation of RNA molecules in the active center cleft of RNAP leading to loss or gain in function. Approximately, 93% of the highly destabilizing mutations at RNA-interacting residues are substitutions to aspartate. Mutations to glutamate were also noted in 6.83% and additionally one each of methionine, proline and threonine mutations indicated highly destabilizing effects.

### 3.6 Substitutions to arginine predominate mutations that destabilize β-subunit-rifampin affinity:

**Fig. 6:**
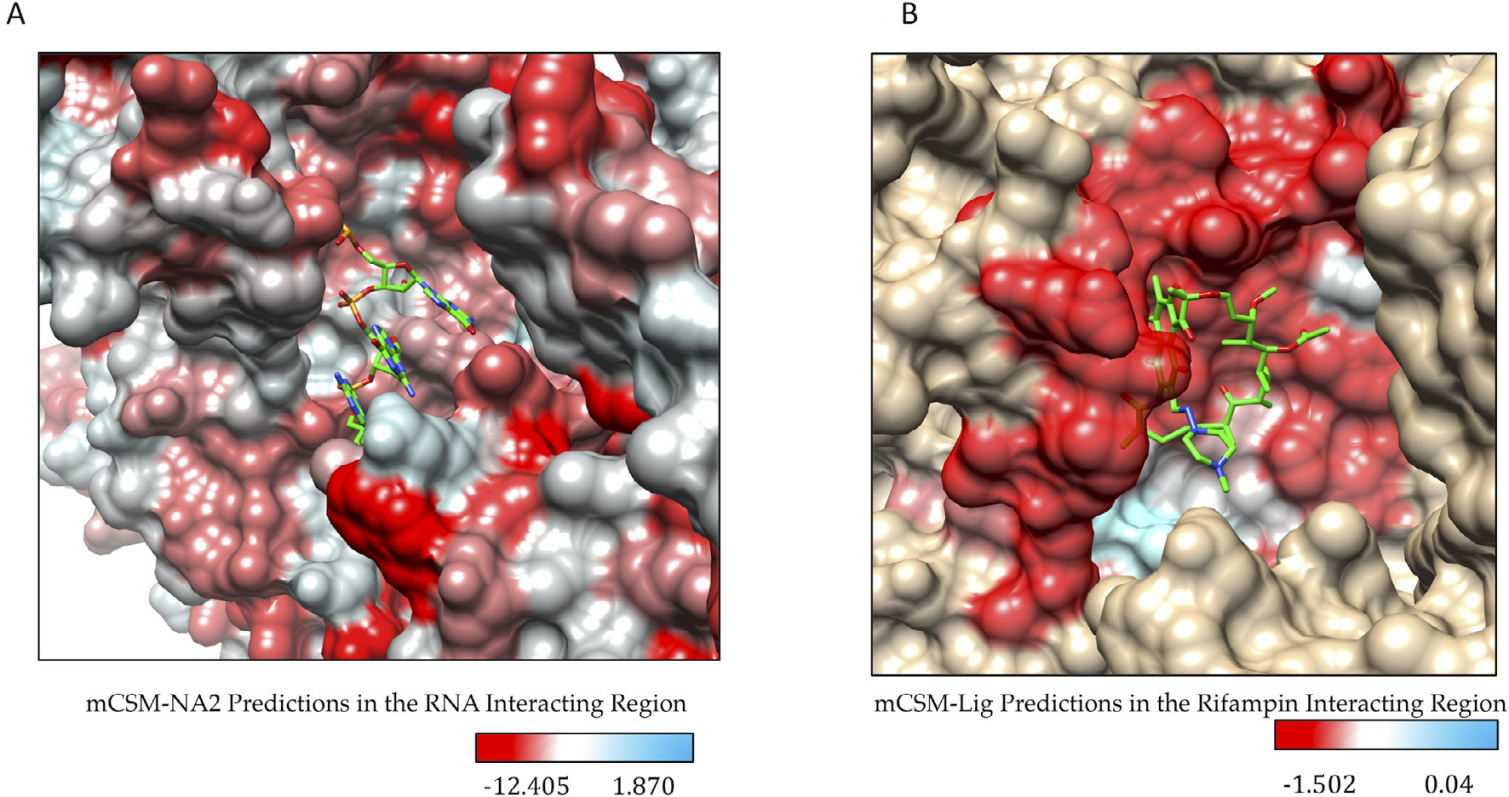
[A] Stability changes in β-subunit -RNA and β-subunit- rifampin [B] interactions due to mutations in the binding sites as predicted by mCSM-NA2 and mCSM-lig. The maximum destabilizing effect a mutation can cause at each residue position in the binding site is depicted on the structure.

Systematic mutations in the set of 70 residues that lie 10 Å from the rifampin binding site reveal that mutations that largely destabilize RNAP-rifampin affinities are primarily arginine and glutamate substitutions (mCSM-lig). In the binding site, R173, R454, R465 and R613 form hydrogen bonds and a network of other interactions with rifampin that stabilize the molecule in the binding site [4]. Introduction of additional arginine residues by mutations may influence the stability and orientation of rifampin in the binding site. The positively charged guanidinium ion of arginine forms cation-π interactions with aromatic amino acids as noted in earlier studies [40], [41]. In the predicted mutations S437R and G456R, arginine forms an intricate network of π interactions with surrounding aromatic amino acids changing the shape of the binding pocket and leading to a loss in rifampin interactions (rifampin retains only two polar contacts with Q438 and F439 whereas wildtype has five hydrogen bonds). The effects of mutations on RNA and rifampin affinity as predicted by mCSM-NA2 and mCSM-lig were mapped on to the structure (Fig. 6A and B).

To determine if mCSM-lig predicted RNAP-rifampin binding affinities can provide information on the degree of resistance associated with each mutation in the rifampin binding site, we attempted to correlate MIC values of *M. tb rpoB* mutants with mCSM-lig predictions for RNAP-rifampin affinity in the structure of *M. tb* (PDB Id: 5UHC). A total of 40 mutations were selected from two studies [12], [42] and mCSM-lig predictions were correlated with MIC values. It was noted that mCSM-lig predictions were independent of the MIC values which was also observed in an earlier study [43]. A table with MIC values and corresponding mCSM-lig predictions was included in Supplementary Material S1 (Table SM1). Additionally, a table with saturated mutations for all residues within 10 Å of the rifampin and their mCSM-lig predictions were presented in Supplementary Material S1 (Table SM2).

### 3.7 Detrimental mutations:

Among all the experimentally identified and computational predicted mutations, we selected those that highly destabilize (maximum decrease in log affinity fold change among all 19 mutations at each residue position) RNAP-rifampin interactions. Six residues were chosen based on the following characteristics and the structural effects of systematic mutations at each residue position were analyzed (Table 2) as below:

• Mutations that highly destabilize rifampin binding (at wildtype S437 & G459 positions) as predicted by mCSM-lig.
• Experimentally/clinically identified and validated mutations that highly destabilize rifampin binding (at wildtype H451 & P489 positions) [9], [10].
• Predicted extremely detrimental mutations for protein stability, protein–protein and protein-nucleic affinities (at wildtype K884 & H1035 positions).

**Table 2:** Detrimental mutations and their corresponding stability changes that influence holoenzyme assembly, rifampin and RNA interactions.

Method	Wild-type residue	Residue position	Average stability effect	Maximum stabilizing effect	Mutant residue	Maximum destabilizing effect	Mutant residue
mCSM-Stability (ΔΔG in kcal/mol)	S	437	−0.795	−0.072	L	−1.701	H
	H	451	−1.214	−0.104	Y	−1.898	S
	G	459	−0.713	−0.381	V	−1.201	W
	P	489	−1.135	−0.507	R	−1.771	G
	K	884	−1.227	−0.190	L	−2.298	S
	H	1035	−0.419	0.600	Y	−1.421	G

mCSM-ppi (ΔΔG in kcal/mol)	S	437	−0.254	0.395	H	−0.820	R
	H	451	−0.652	−0.050	S	−1.451	M
	G	459	−0.397	0.237	H	−1.042	R
	P	489	−0.738	−0.138	W	−1.372	R
	K	884	−0.105	0.160	D	−0.685	R
	H	1035	−0.754	0.115	W	−1.726	R

mCSM-NA2 (ΔΔG in kcal/mol)	S	437	−1.538	4.922	W	−3.857	D
	H	451	−1.300	5.147	W	−3.632	D
	G	459	2.289	8.556	W	−0.221	D
	P	489	1.926	8.195	W	−0.582	D
	K	884	0.221	6.647	W	−2.130	D
	H	1035	0.847	7.295	W	−1.484	D

mCSM-lig (log-affinity change)	S	437	−0.646	−0.484	L	−1.062	R
	H	451	−0.510	−0.076	W	−0.777	E
	G	459	−0.981	−0.715	A	−1.236	R
	P	489	−0.598	−0.254	L	−0.917	R
	K	884	−0.156	−0.368	D	−0.925	R
	H	1035	−0.121	0.097	V	−0.501	E

SDM (ΔΔG in kcal/mol)	S	437	0.087	2.320	V	−1.900	P
	H	451	−0.756	1.290	L	−2.800	G
	G	459	−2.842	−1.780	V	−3.800	P
	P	489	−0.432	1.440	Y	−1.070	E
	K	884	0.108	1.270	V	−1.820	P
	H	1035	−0.200	0.590	V	−1.410	P

MAESTRO (ΔΔG in kcal/mol)	S	437	−0.21	−0.14	K	0.24	F
	H	451	−0.12	−0.05	G	0.22	R
	G	459	−0.23	−0.17	S	0.33	W
	P	489	−0.26	−0.22	H	0.31	M
	K	884	−0.20	−0.14	G	0.25	M
	H	1035	−0.27	−0.25	P	0.31	Y

CUPSAT (ΔΔG in kcal/mol)	S	437	2.70	7.98	I	−1.12	G
	H	451	2.01	6.92	W	−3.25	K
	G	459	−2.51	5.00	K	−5.53	C
	P	489	−2.76	−0.84	A	−5.47	M
	K	884	−2.99	3.42	I	−8.03	H
	H	1035	−1.07	2.15	C	−3.23	Y

Imutant 2.0 Structure (Sign of prediction)	S	437	4.05	9.00	A	1.00	F
	H	451	6.00	8.00	G	3.00	L
	G	459	6.63	9.00	N	3.00	I
	P	489	7.11	9.00	G	3.00	L
	K	884	6.42	9.00	G	2.00	M
	H	1035	4.63	8.00	G	2.00	L

PROVEAN (ΔΔG in kcal/mol)	S	437	−4.79	−3.00	A	−7.00	W
	H	451	−8.66	−5.73	Y	−10.37	C
	G	459	−8.10	−6.00	A	−10.00	L
	P	489	−9.04	−7.99	A	−10.99	F
	K	884	−5.97	−2.91	R	−7.75	C
	H	1035	−8.98	−5.79	Y	−10.61	C

Imutant 2.0 Sequence (Sign of prediction)	S	437	4.47	7.00	F	0.00	H
	H	451	3.21	7.00	P	0.00	F
	G	459	3.53	7.00	H	0.00	A
	P	489	6.89	9.00	G	5.00	L
	K	884	3.53	8.00	V	0.00	G
	H	1035	2.95	6.00	G	0.00	V

FOldX4 (ΔΔG in kcal/mol)	S	437	2.79	−1.44	I	12.39	R
	H	451	1.78	−0.74	L	4.39	W
	G	459	9.14	3.96	A	20.76	H
	P	489	3.04	2.11	N	4.79	R
	K	884	1.06	−2.12	Y	9.77	L
	H	1035	0.77	−1.47	P	5.69	Y

ENCoM (ΔΔSvib in kcal/mol/K)	S	437	−0.44	0.48	G	−1.50	W
	H	451	0.34	0.97	G	−0.46	W
	G	459	−0.91	−0.29	A	−1.55	W
	P	489	−0.16	0.14	G	−0.82	F
	K	884	0.18	0.96	G	−0.60	W
	H	1035	0.19	0.73	G	−0.26	W

DynaMut (ΔΔG in kcal/mol)	S	437	2.87	6.99	L	−2.08	G
	H	451	−0.74	2.17	Y	−3.43	T
	G	459	1.93	3.29	N	−0.25	S
	P	489	0.94	3.26	F	−0.72	S
	K	884	0.14	3.69	W	−1.87	E
	H	1035	0.21	2.38	W	−2.29	G

### 3.8 Detrimental mutations in the rifampin binding site:

We have noted that any mutation at rifampin-interacting residues S437, H451, R454, S456, L458, G459, R465, P489, P492 and N493 destabilize protein ligand affinity (mCSM-lig). Of these we have chosen wild-type residues H451 and P489, which are experimentally identified mutations, and wild-type residues S437 and G459, which are computationally predicted (only one mutation was experimentally identified at residue position S437L (reported by us earlier [4], and has destabilizing effects on the overall stability of the protein and affinity to rifampin).

### 3.9 S437

**Fig. 7:**
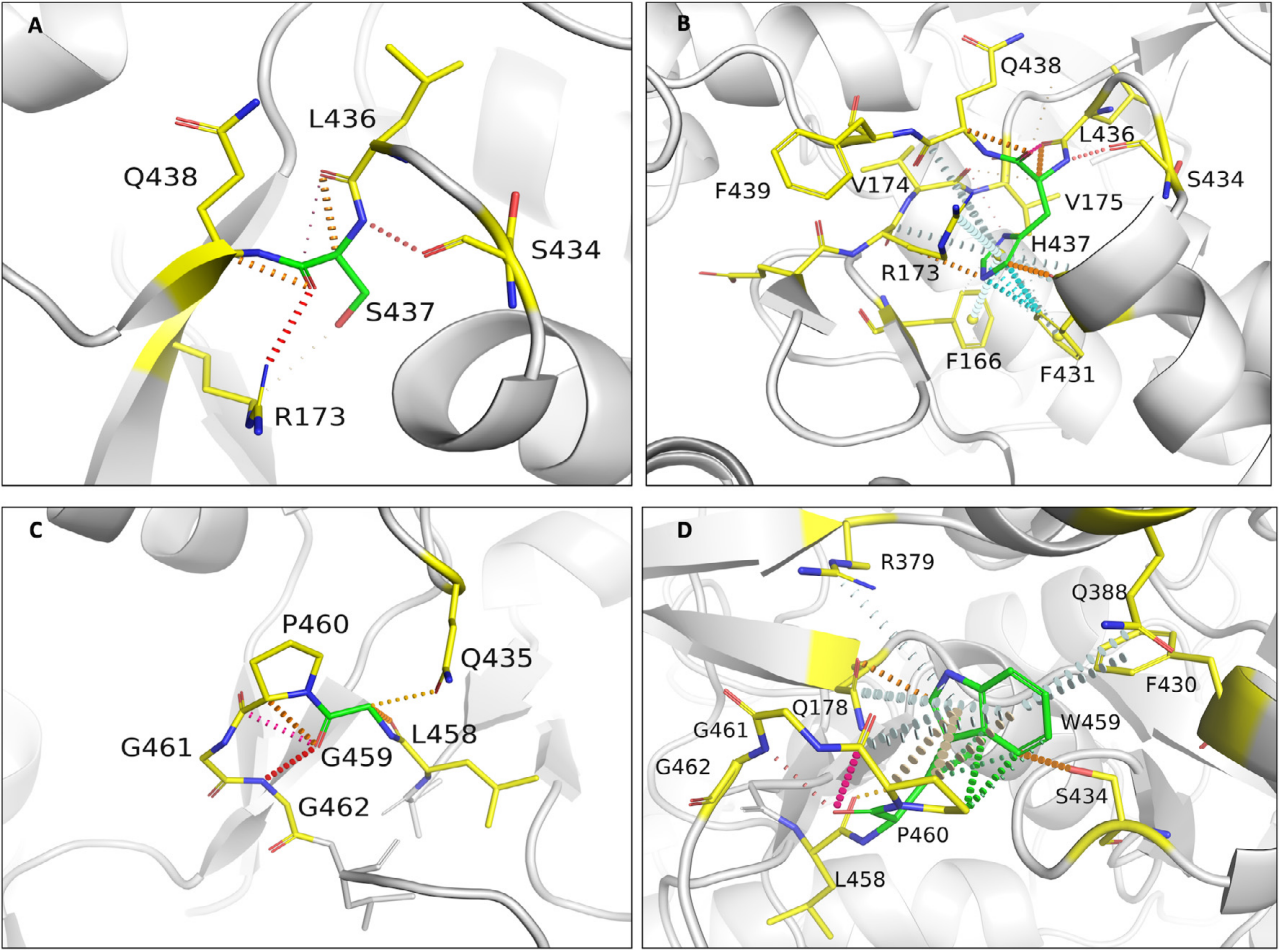
[A] Interactions of S437 with the surrounding residue environment in the wildtype and of H437 in the S437H mutant [B]. [C] Interactions of G459 with the surrounding residue environment and [D] W459 in the mutant G459W. The red dotted lines represent hydrogen bonds. Orange dotted lines represent weak hydrogen bond interactions. Ring-Ring and intergroup interactions are depicted in cyan. Aromatic interactions are represented in sky-blue and carbonyl interactions in pink dotted lines. Green dotted lines represent hydrophobic interactions. (For interpretation of the references to colour in this figure legend, the reader is referred to the web version of this article.)

Serine at position 437 in the wild-type structure forms mainchain and sidechain hydrogen bonds with S434, G432 and R173. The residue has a network of proximal polar interactions and hence stabilizes the rifampin-binding pocket. It was noted that any mutation at this position reduces rifampin affinity (mCSM-lig) and stability of the β-subunit (mCSM) (Supplementary Table 1) (Fig. 7A). The maximum destabilizing effect was noted for substitution to histidine (−1.701 kcal/mol (mCSM)) and it forms hydrogen bonds with S434 and Q438, aromatic bonds with F431, and many ring-ring and π interactions with the surrounding residues which might largely effect the shape of the binding pocket (Fig. 7B). Substitution with leucine causes a minimal destabilizing effect (−0.072 kcal/mol (mCSM)) and stability effects of all the other amino acid substitutions range from −0.072 to −1.701 kcal/mol (mCSM).

S437 is located at 3.3 Å from the interface of β and β′ subunits. Arginine substitution destabilized the interface with the predicted stability change of −0.820 kcal/mol (mCSM-ppi). In the wild-type structure, S437 is located 11.9 Å from the closest nucleic acid molecule but is present on the helix that interacts with both DNA and transcribing RNA in the active center cleft. An aspartate substitution destabilized the protein-RNA interaction with predicted affinity change of −3.857 kcal/mol (mCSM-NA2). S437 is located 4.0 Å from rifampin and forms only proximal interactions with rifampin. However, this residue forms hydrogen bond interactions with S434 and R173 that are important for the attachment of rifampin to the binding pocket. The S437R mutation disrupts the hydrogen bonds with S434 and R173 which in-turn impact stability of rifampin in the binding pocket (−1.062 kcal/mol (mCSM-lig)).

### 3.10 G459

Glycine at position 459 forms hydrogen bonds with Q435, L458 and G462, and carbonyl interactions with the P460. G459 is present 4.6 Å away from rifampin and is involved in hydrogen bonds with residues that interact with rifampin (Fig. 7C). A tryptophan substitution largely destabilizes the binding pocket by the incorporation of hydrophobic and π interactions with the surrounding residues. It forms side-chain hydrophobic interactions with L436, L384 and F430. It also forms a ring–ring interaction with F430, an atom-ring interaction with L384 and intergroup interactions with Q178 and Q388. It forms multiple hydrogen bonds with the surrounding residues, which may impact the orientation of the binding pocket and destabilize the protein (Fig. 7D).

### 3.11 Clinically identified mutations that highly destabilize rifampin binding:

From the 40 mutations that are reported from different rifampin-resistant leprosy clinical isolates (Supplementary Table 2), we have chosen two residues where mutations are extremely detrimental to protein stability, protein ligand affinity, protein nucleic affinity and protein subunit interfaces. These substitutions at positions H451 and P489 were studied in detail.

### 3.12 H451:

**Fig. 8:**
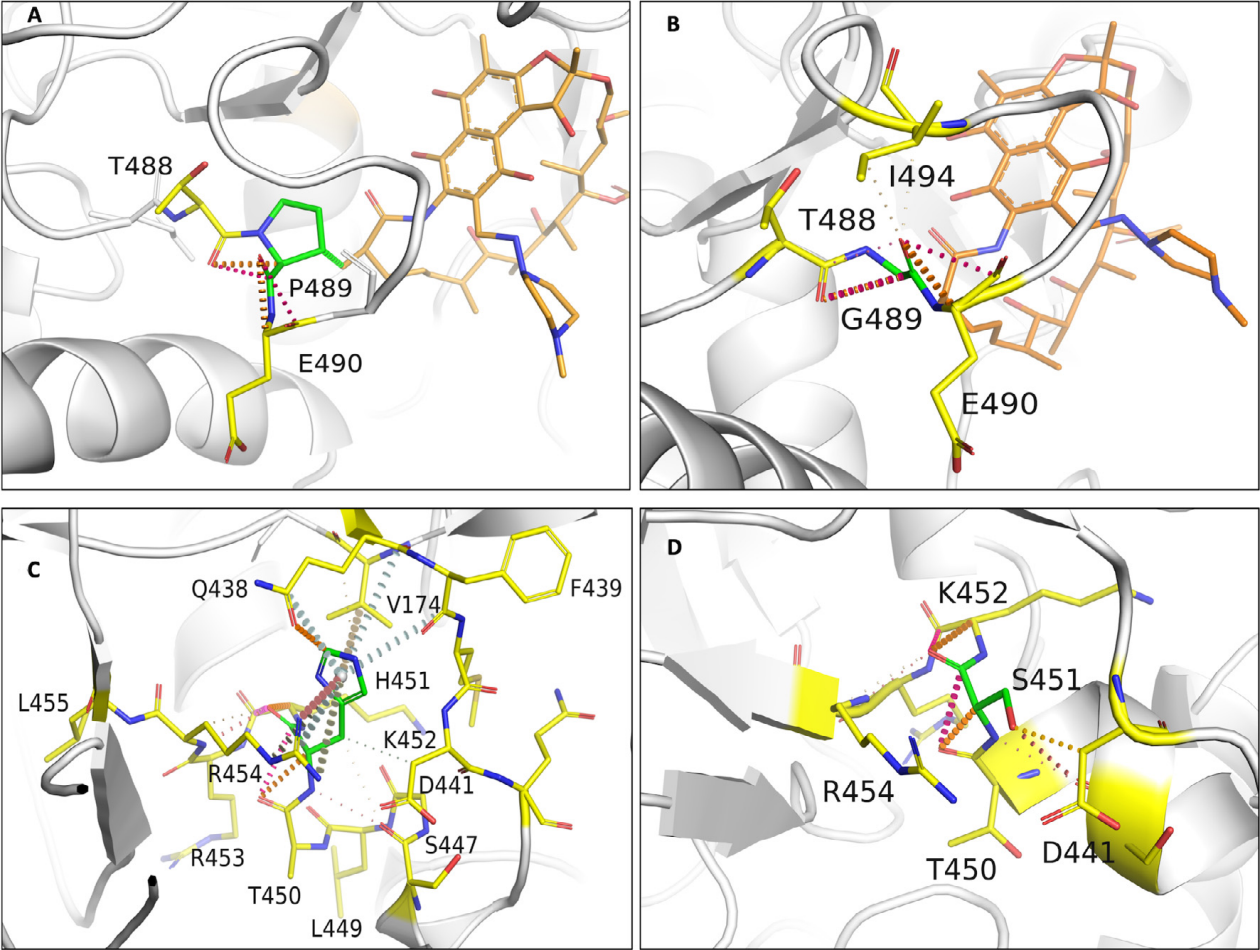
[A] Interactions of P489 with the surrounding residue environment in the wildtype and of G489 in the P489G mutant [B]. [C] Interactions of H451 with the surrounding residue environment and [D] S451 in the mutant H451S.

H451 in the wild-type structure lies 3.7 Å from rifampin and 4.1 Å from the interface. This residue forms cation – π interactions with guanidinium group of R454, which in turn forms polar interactions with rifampin (Fig. 8A). Additionally, H451 makes two hydrogen bonds with mainchain amino group of R454 and oxygen atom of S447. Mutations at this residue site largely impact the stability and ligand binding. Substitution to serine induced a change in stability of the protein with a decrease in Gibbs free energy of −1.898 kcal/mol and a network of π interactions that are present in the native structure, were lost in the mutant (Fig. 8B).

Methionine substitution destabilizes β – β′ subunit interface and leads to a change in free energy of −1.451 Kcal/mol. Methionine forms carbonyl interactions with K452 and T450, a hydrophobic interaction with Q438 and weak hydrogen bond interactions with rifampin. Although histidine or methionine do not directly interact with the residues of the β′ subunit, the changes in the network of π-interactions coupled with the addition of hydrophobic bonds among proximal residues in the interface may change their binding patterns leading to destabilization of the interface.

Substitution with glutamic acid induces a destabilizing effect on the β-subunit-rifampin interaction. E451 forms weak hydrogen bond, carbonyl and proximal hydrophobic interactions with the residue environment but does not form any bonds with rifampin, unlike the wild-type residue that forms proximal hydrogen bonds with rifampin.

### 3.13 P489

Proline at position 489 is present in a loop which is in close proximity to rifampin and forms hydrophobic interaction with rifampin and weak hydrogen bond interactions with T488 and Q490 (Fig. 8C). Mutations at the position 489 were reported in rifampin-resistant leprosy patients from Thailand [9]. Glycine substitution destabilizes the protein (−1.771 kcal/mol) leading to a loss of hydrophobic interaction with rifampin. Weak hydrogen bond and carbonyl interactions, however, were retained in the mutant model (Fig. 8D). Arginine substitution destabilizes interface and rifampin affinities, with predicted stability changes of −1.372 and −0.917 kcal/mol respectively. FoldX predicted a large change in stability of 4.79 kcal/mol for difference between mutant and wild types, which is highly destabilizing. FoldX optimizes the sidechains and moves the structure to a lowest energy state (usually represented as a negative value) and hence the difference between two negative energy values of wild and mutant is considered destabilizing.

### 3.14 Extremely detrimental mutations:

Mutations at residues positions K884 and H1035 were considered to be extremely detrimental. These residues lie in close proximity to the interface, nucleic acids and rifampin. Substitutions at these sites destabilize protomer, protein–protein interfaces (both the residues reside at the subunit interface), protein-nucleic acid and protein–ligand affinities. Both empirical (FoldX) and knowledge based (mCSM and SDM) methods predicted destabilizing effects.

### 3.15 K884

**Fig. 9:**
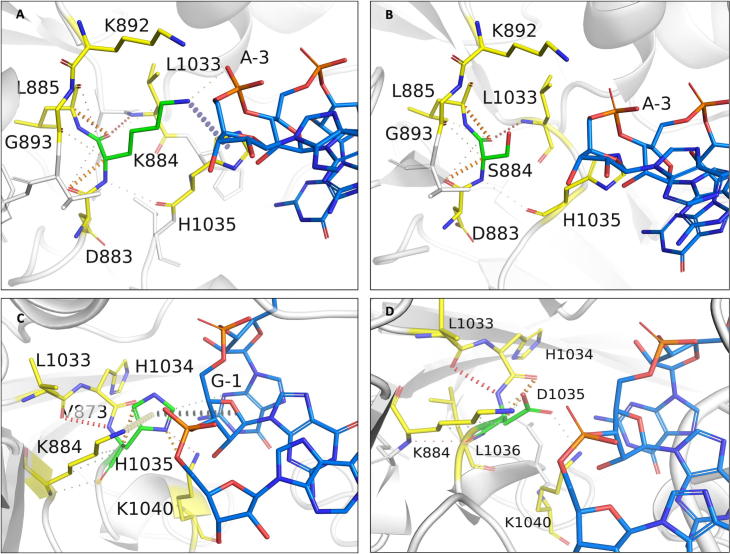
[A] Interactions of K884 with the surrounding residue environment in the wildtype and of S884 in the K884S mutant [B]. [C] Interactions of H1035 with the surrounding residue environment and [D] D1035 in the mutant H1035D. The blue dotted lines represent cation-π interaction. (For interpretation of the references to colour in this figure legend, the reader is referred to the web version of this article.)

K884 is located 3.2 Å from the interface, 3.3 Å from the nucleic acid and 8.6 Å from rifampin. Lysine forms mainchain hydrogen bonds with L1033 and proximal hydrophobic interactions with H1035 and V894. It also forms a cation - π interaction with H1035 and most importantly a sidechain proximal hydrogen bond with the sugar phosphate group of guanine (second) nucleotide in the RNA transcript. This interaction is crucial for maintaining the RNA interaction with rifampin in order to induce steric clash on the adjacent nucleotide and halt transcription (Fig. 9A). Serine substitution at this site results in the loss of this vital interaction. S884 forms weak Van der Waals interactions with D883 and L885 and hydrogen bonds with L1033 and H1035. Interactions with RNA backbone are lost in the mutant (Fig. 9B). The mutant is destabilized (−2.298 kcal/mol).

Aspartate substitution at this site destabilizes RNA affinity (−2.130 kcal/mol) and the mutant residue forms hydrogen bonds with L1033 and H1035, and hydrophobic interactions with V894.

### 3.16 H1035

Histidine at position 1035 is located 3.5 Å from the interface and RNA, and 8.8 Å away from rifampin. It forms a network of π interactions with the surrounding residues. The ring-ring π interactions with the fused pyrimidine-imidazole ring of guanine in the first nucleotide of RNA transcript is vital to the orientation of RNA transcript in the active center cleft (Fig. 9C). These interactions are lost in substitutions with non-aromatic amino acids. It was also noted that aspartate substitution largely destabilizes β subunit -rifampin affinity (Fig. 9D).

### 3.17 Impact of mutations on flexible conformations:

**Fig. 10:**
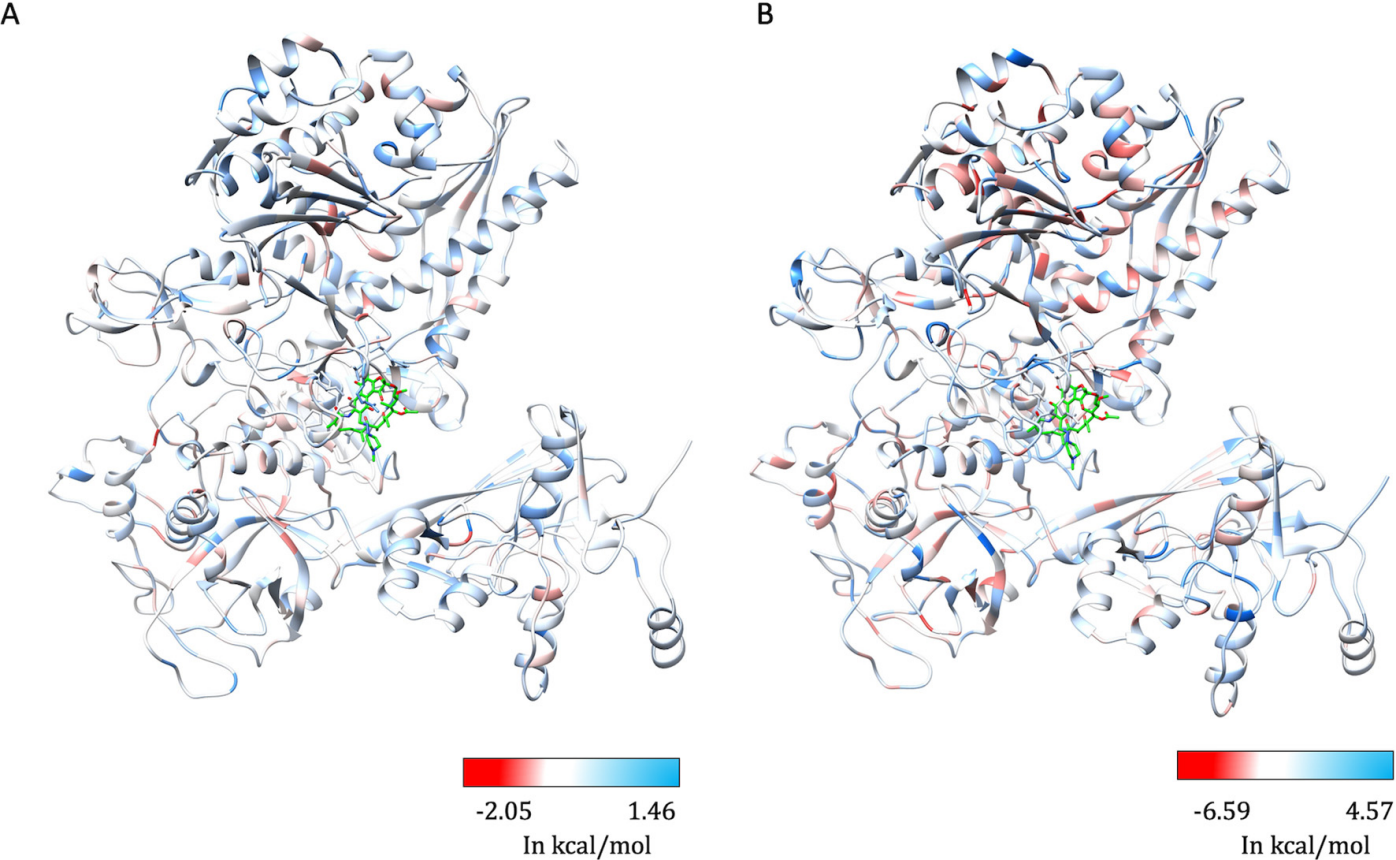
[A] The maximum destabilizing effects on the protein stability, a mutation can induce at each residue position in the flexible conformations (as predicted by ENCoM [A] and DynaMut [B]), are mapped on the structure. Regions in red represent highly destabilizing while the blue regions are relatively stable with mutations. (For interpretation of the references to colour in this figure legend, the reader is referred to the web version of this article.)

The stability changes between the wildtype and each mutant in lowest energy conformation were calculated by FoldX and have a Pearson’s correlation coefficient (“r” value) of 0.38 with other predictors mCSM and SDM. Although FoldX does not probe backbone conformational changes, it optimizes the sidechain rotamers of the mutant residues to attain a low energy state and calculates the change in free energy between the states. We further sampled the fully flexible conformers of the β-subunit and estimated changes in vibrational entropy ΔS and protein stability using ENCoM. A linear combination of vibrational entropy ΔS by ENCoM and enthalpy changes by FoldX were used to calculate stability changes. ENCoM predicted highly destabilizing mutations in the rifampin binding and RNA interacting sites in the active center cleft of the holoenzyme. DynaMut predictions correlated with ENCoM values at an r value of 0.56. The average change in stability predicted by ENCoM and DynaMut for any mutation at each residue position in the β subunit was mapped on the model (Fig. 10A and B).

### 3.18 Protein stability changes and fragment hotspot maps:

**Fig. 11:**
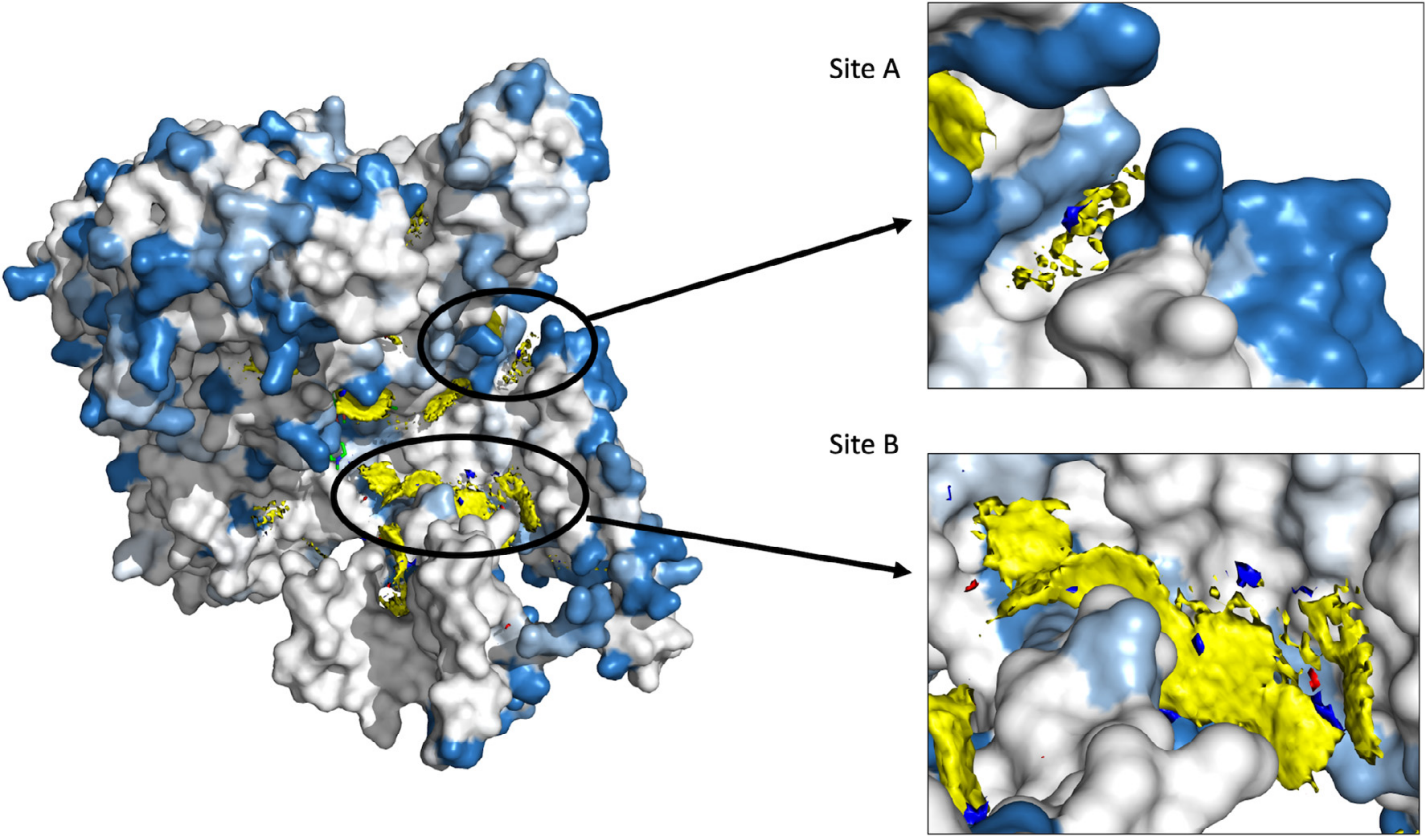
Fragment hotspots were mapped on the structure which was coloured with maximum destabilizing effects of systematic mutations at each residue positions. Blue represents regions which are least impacted by any mutations. Stable and potential small molecule binding sites “A” and “B” are depicted on the structure.

Fragment hotspots were mapped on the structure that is colored by regions predicted to have least protein stability changes due to any mutations (using mCSM, SDM and FoldX software). As fragment hotspot maps program identifies small molecule binding propensity on the surface of the protein, we used only the protein stability prediction software to identify areas that are stable by any mutations. The regions of the β subunit that are least impacted by mutations (mutation coolspots) are overlaid with fragment hotspot maps. The site B (Fig. 11), which is in close proximity to the RNA binding region and is a pocket at the β-β′ subunit interface, is least impacted by mutations and has a hotspot at the contouring score of 17 with donor, apolar and acceptor regions [22]. Secondly, the site A, although located away from the catalytic core of the enzyme, is present in the path of entry/exit point for template DNA into the holoenzyme complex and a small molecule interaction at this site can potentially impact template DNA interactions or induce conformational change in the crab-claw-shaped β subunit leading to disruption in the holoenzyme assembly.

## 4 Discussion

In the absence of a rapid and an effective laboratory-based diagnostic tool for determining drug resistance in leprosy, identification of mutations known to confer resistance to individual drugs in MDT remains an appropriate approach for diagnosing drug resistance. Associations between mutations in the drug targets and clinical resistance to individual drugs in MDT are often validated by mouse-footpad experiments in which, resistant strains (with known mutations) are propagated in the hind footpads of mice (cross-bred albino) in the presence of drugs under study [4]. Owing to high percentage identity of the β subunit of RNAP of *M. leprae* with that of *M. tuberculosis,* identical mutations that are experimentally proven to confer rifampin resistance in tuberculosis, are considered as likely drug-resistant mutations in leprosy. The experimentally known mutations in *M. leprae* were those identified by DNA sequencing of *rpoB* gene (derived from skin tissue DNA of relapsed/drug resistant leprosy patients) and published in different studies (reference for each mutation is listed in Supplementary Table 2). Most of these were validated in either mouse foot-pad experiments or by using surrogate genetic hosts [5].

Around 40 different rifampin-resistance mutations were noted in *M. leprae* from clinical isolates around the world using amplicon sequencing of RRDR [10]. All of these mutations decrease the stability of rifampin binding to the β-subunit (Supplementary Table 2) and the mutant strains exhibited normal grown patterns in the mouse footpads when administered with rifampin in doses equivalent to WHO regimen of multibacillary MDT [44]. This indicates that mutations structurally and functionally impact rifampin interactions and influence concomitant resistance.

Thermodynamic stability of the proteins essentially influences their function and is largely dependent on the sequence. Missense mutations that lead to amino acid substitutions often impact protein stability, shifting it towards either a stabilized or a destabilized state [7]. Experimental measurements of stability changes in proteins are often challenging especially with large and complex protein machineries like RNAP. However, mutations within each subunit of the RNAP complex, and primarily the rifampin binding β-subunit, have clinical implications and influence rifampin-resistance outcomes in mycobacterial diseases [45]. The performance of various structural, sequence and NMA based predictors for predicting protein stability changes upon mutations vary largely in terms of their accuracy and bias [46], but offer a quick and a helpful alternative to understanding the association between mutations and resistance phenotypes [6].

Given the absence of a rapid and experimentally validated system to read the impact of mutations in the β-subunit of RNAP in *M. leprae* with clinical rifampin resistance outcomes in leprosy, we conducted computational saturation mutagenesis to determine regions on the β-subunit that impact the overall stability, protein-subunit interfaces, protein-nucleic and protein–ligand affinities. Being a part of the complex transcriptional machinery in the mycobacterial cell, the compositional and conformational stability of the β-subunit is crucial to binding of DNA template and synthesis of complementary RNA transcript in the active center cleft of the holoenzyme [47], [48]. As rifampin blocks the growing RNA transcript through steric occlusion, its binding and orientation in the binding pocket is vital to its function [47]. Mutations within the RRDR impact rifampin interactions and overall stability of the subunit. As noted from Supplementary Table 2, all the experimentally identified *rpoB* gene mutations from *M. leprae* indicated a destabilizing effect on the protein–ligand affinity. Owing to the robustness of these predictions, we employed an *in-silico* saturation mutagenesis model to understand the impacts of systematic mutations at each residue site of the subunit.

The destabilizing mutations are given preference over mutations that are silent or have minimal effects on the stability. This is to explore and understand the possible structural and functional implications of emerging detrimental mutations (reported or new) that can influence rifampin resistance outcomes in leprosy. We used different structural, sequence and NMA based tools to identify and compare the predictions. mCSM stability predictions had better correlations with the other predictors (SDM (r = 0.55), MAESTRO (r = 0.61), Imutant 2.0 Structure (r = 0.72), CUPSAT (r = 0.43), Imutant 2.0 Sequence (r = 0.62) and DynaMut (r = 0.61)).

Protocols (Computational Saturation Mutagenesis (CoSM)) [49] that use molecular dynamic equilibration, sidechain flips and energy minimization to improve side conformations in mutants enable prediction of stability changes with better accuracy and correlation with the experimentally deciphered stability changes (r = 0.9). However, these protocols are computationally intensive and require high performance computing systems and time. CoSM had a similar performance to FoldX, which was used in the current study. Given the large sample size, molecular dynamic equilibration of sidechain rotamers is beyond the scope of this study.

In conclusion, we have deciphered the predicted effects of all possible mutations in the β-subunit of RNAP in *M. leprae* using computational saturation mutagenesis model, probing structural, sequence driven and dynamic changes that impact overall stability of the protein, RNA and rifampin affinities. The predicted impacts were mapped onto the structures and highly detrimental mutations were further analyzed for their changes in interatomic interactions. Due to the lack of adequate experimental data on stability changes in β-subunit of RNAP upon mutations, we have limited information on the accuracy of the predictions, however, all the prediction tools used in the study are well tested and validated software which are proven to perform with reasonable accuracy and minimal bias on various relevant mutational datasets [31]. To date there were no studies describing the phenotypic resistance/susceptibility outcomes in strains with compensatory mutations in RNAP. Further studies on saturation mutagenesis of the entire RNAP holoenzyme complex may provide comprehensive information on the effects of co-evolving and compensatory mutations in other subunits on rifampin binding and function.

## Declaration of Competing Interest

The authors declare that they have no known competing financial interests or personal relationships that could have appeared to influence the work reported in this paper.
