# Supplementary material for: Computational saturation mutagenesis to predict structural consequences of systematic mutations in the beta subunit of RNA polymerase in Mycobacterium leprae
Source: Comput Struct Biotechnol J. 2020 Jan 17;18:271–86. doi: 10.1016/j.csbj.2020.01.002 (PMC7000446; doi:10.1016/j.csbj.2020.01.002)
Supplement: Supplementary data 1 [file mmc1.docx]

**Supplementary Material S1**

**Title:** Computational Saturation Mutagenesis to predict structural consequences of systematic mutations in the beta subunit of RNA polymerase in *Mycobacterium leprae.*

**Running Title:** *Computational* *Saturation Mutagenesis–RpoB M. leprae*

**Authors:** Sundeep Chaitanya Vedithi^1*^, Carlos H. M. Rodrigues^2,3^, Stephanie Portelli^2,3^, Marcin J. Skwark^1^, Madhusmita Das^4^, David B. Ascher^1,2,3^, Tom L Blundell^1*^ & Sony Malhotra^1,5^

**Table SM1:** Comparison of MICs in *M. tuberculosis (M. tb)* with the mCSM-lig predictions of RNAP-Rif affinity in *M. tb.*

| **Mutation in *M. tb rpoB gene**** | **MIC for Mutations  in Tuberculosis in ug/ml** | ***M. tb* Phenotype** | **Pubmed ID** | **mCSM-lig predictions in PDB id: 5UHC (chain C) in  log (Affinity Fold Change)** |
| --- | --- | --- | --- | --- |
| F430L | 100.00 | Highly Resistant | 19209951 | -0.56 |
| G432S | 50.00 | Moderately Resistant | 19209951 | -0.79 |
| G432D | 100.00 | Highly Resistant | 19209951 | -0.79 |
| T433A | 75.00 | Moderately Resistant | 19209951 | -0.54 |
| T433S | 128.00 | Highly Resistant | 19209951 | -0.34 |
| T433P | 50.00 | Moderately Resistant | 19209951 | -0.52 |
| T433H | 100.00 | Highly Resistant | 19209951 | -0.56 |
| F439L | 12.50 | Low Resistance | 19209951 | -0.62 |
| D441H | 75.00 | Moderately Resistant | 19209951 | -0.44 |
| N444K | 50.00 | Moderately Resistant | 19209951 | -0.13 |
| L446P | 0.39 | Susceptible | 19209951 | 0.17 |
| S447L | 8.00 | Low Resistance | 19209951 | 0.18 |
| S447Q | 256.00 | Highly Resistant | 19209951 | 0.24 |
| G448A | 100.00 | Highly Resistant | 19209951 | 0.18 |
| G448W | 128.00 | Highly Resistant | 19209951 | -0.36 |
| T450I | 128.00 | Highly Resistant | 19209951 | 0.53 |
| H451P | 8.00 | Low Resistance | 19209951 | -0.58 |
| H451F | 100.00 | Highly Resistant | 19209951 | -0.29 |
| H451Q | 8.00 | Low Resistance | 19209951 | -0.73 |
| K452N | 40.00 | Moderately Resistant | 19209951 | 0.23 |
| R453P | 64.00 | Moderately Resistant | 19209951 | 0.03 |
| R453H | 128.00 | Highly Resistant | 19209951 | 0.08 |
| S456Y | 8.00 | Low Resistance | 19209951 | -0.46 |
| S456W | 8.00 | Low Resistance | 19209951 | -0.50 |
| L458P | 32.00 | Moderately Resistant | 19209951 | -1.10 |
| E466G | 128.00 | Highly Resistant | 19209951 | -0.83 |
| S478A | 128.00 | Highly Resistant | 19209951 | -0.01 |
| E487G | 1.00 | Low Resistance | 19209951 | -0.12 |
| P489L | 1.00 | Low Resistance | 19209951 | -0.72 |
| I497F | 256.00 | Highly Resistant | 19209951 | -0.76 |
| S499L | 64.00 | Moderately Resistant | 19209951 | 0.40 |
| V152F | 50 | Moderately Resistant | 24740074 | -0.87 |
| D441A | 160 | Highly Resistant | 24740074 | -0.38 |
| D441V | 50 | Moderately Resistant | 24740074 | -0.40 |
| H451D | 160 | Highly Resistant | 24740074 | -0.79 |
| H451L | 2 | Low Resistance | 24740074 | -0.42 |
| H451Y | ≥160 | Highly Resistant | 24740074 | -0.26 |
| S447L | 20 | Moderately Resistant | 24740074 | 0.18 |
| S456L | 160 | Highly Resistant | 24740074 | -0.39 |
| S456W | ≥160 | Highly Resistant | 24740074 | -0.50 |

*The numbering of the residue positions has been changed to match with the residue numbering in RNAP structure (PDB Id: 5UHC) of *M. tb*.

Table SM2 on the next page…

**Table SM2:** Table with saturated mutations of all the residues within 10 Å of the rifampin with mCSM-lig predicted RNAP-rifampin affinities:

| **Mutations within the Rifampin binding site in the β subunit of RNAP in *M. leprae*** | **mCSM-lig Prediction of log change in RNAP-Rifampin affinity** | **Distance from Rifampin in Å** | **mCSM-lig Effect** |
| --- | --- | --- | --- |
| T171G | -0.101 | 8.542 | Destabilizing |
| T171A | -0.045 | 8.542 | Destabilizing |
| T171S | -0.351 | 8.542 | Destabilizing |
| T171C | -0.594 | 8.542 | Destabilizing |
| T171L | 0.012 | 8.542 | Stabilizing |
| T171M | -0.292 | 8.542 | Destabilizing |
| T171P | -0.015 | 8.542 | Destabilizing |
| T171F | -0.171 | 8.542 | Destabilizing |
| T171Y | -0.16 | 8.542 | Destabilizing |
| T171W | -0.304 | 8.542 | Destabilizing |
| T171D | -0.622 | 8.542 | Destabilizing |
| T171E | -0.61 | 8.542 | Destabilizing |
| T171N | -0.358 | 8.542 | Destabilizing |
| T171Q | -0.342 | 8.542 | Destabilizing |
| T171H | -0.41 | 8.542 | Destabilizing |
| T171K | -0.487 | 8.542 | Destabilizing |
| T171R | -0.733 | 8.542 | Destabilizing |
| E172G | -0.115 | 8.02 | Destabilizing |
| E172A | -0.123 | 8.02 | Destabilizing |
| E172T | -0.226 | 8.02 | Destabilizing |
| E172S | -0.221 | 8.02 | Destabilizing |
| E172C | -0.426 | 8.02 | Destabilizing |
| E172V | -0.172 | 8.02 | Destabilizing |
| E172L | -0.202 | 8.02 | Destabilizing |
| E172I | -0.219 | 8.02 | Destabilizing |
| E172M | -0.384 | 8.02 | Destabilizing |
| E172P | -0.139 | 8.02 | Destabilizing |
| E172F | -0.238 | 8.02 | Destabilizing |
| E172Y | -0.266 | 8.02 | Destabilizing |
| E172W | -0.362 | 8.02 | Destabilizing |
| E172D | 0.139 | 8.02 | Stabilizing |
| E172N | -0.168 | 8.02 | Destabilizing |
| E172Q | -0.125 | 8.02 | Destabilizing |
| E172H | -0.317 | 8.02 | Destabilizing |
| E172K | -0.286 | 8.02 | Destabilizing |
| E172R | -0.43 | 8.02 | Destabilizing |
| R173G | -0.024 | 4.268 | Destabilizing |
| R173A | 0.077 | 4.268 | Stabilizing |
| R173T | 0.244 | 4.268 | Stabilizing |
| R173S | 0.178 | 4.268 | Stabilizing |
| R173C | -0.177 | 4.268 | Destabilizing |
| R173V | 0.154 | 4.268 | Stabilizing |
| R173L | 0.181 | 4.268 | Stabilizing |
| R173I | 0.181 | 4.268 | Stabilizing |
| R173M | -0.119 | 4.268 | Destabilizing |
| R173P | 0.175 | 4.268 | Stabilizing |
| R173F | 0.026 | 4.268 | Stabilizing |
| R173Y | 0.155 | 4.268 | Stabilizing |
| R173W | -0.044 | 4.268 | Destabilizing |
| R173D | -0.107 | 4.268 | Destabilizing |
| R173E | -0.092 | 4.268 | Destabilizing |
| R173N | 0.29 | 4.268 | Stabilizing |
| R173Q | 0.259 | 4.268 | Stabilizing |
| R173H | 0.253 | 4.268 | Stabilizing |
| R173K | 0.364 | 4.268 | Stabilizing |
| V174G | 0.388 | 7.173 | Stabilizing |
| V174A | 0.573 | 7.173 | Stabilizing |
| V174T | 0.235 | 7.173 | Stabilizing |
| V174S | 0.201 | 7.173 | Stabilizing |
| V174C | 0.03 | 7.173 | Stabilizing |
| V174L | 0.664 | 7.173 | Stabilizing |
| V174I | 0.674 | 7.173 | Stabilizing |
| V174M | 0.285 | 7.173 | Stabilizing |
| V174P | 0.429 | 7.173 | Stabilizing |
| V174F | 0.3 | 7.173 | Stabilizing |
| V174Y | 0.217 | 7.173 | Stabilizing |
| V174W | -0.07 | 7.173 | Destabilizing |
| V174D | -0.313 | 7.173 | Destabilizing |
| V174E | -0.34 | 7.173 | Destabilizing |
| V174N | 0.116 | 7.173 | Stabilizing |
| V174Q | 0.163 | 7.173 | Stabilizing |
| V174H | 0.084 | 7.173 | Stabilizing |
| V174K | 0.182 | 7.173 | Stabilizing |
| V174R | -0.041 | 7.173 | Destabilizing |
| V175G | -0.147 | 8.486 | Destabilizing |
| V175A | -0.069 | 8.486 | Destabilizing |
| V175T | -0.219 | 8.486 | Destabilizing |
| V175S | -0.203 | 8.486 | Destabilizing |
| V175C | -0.475 | 8.486 | Destabilizing |
| V175L | -0.218 | 8.486 | Destabilizing |
| V175I | -0.214 | 8.486 | Destabilizing |
| V175M | -0.462 | 8.486 | Destabilizing |
| V175P | -0.197 | 8.486 | Destabilizing |
| V175F | -0.444 | 8.486 | Destabilizing |
| V175Y | -0.484 | 8.486 | Destabilizing |
| V175W | -0.646 | 8.486 | Destabilizing |
| V175D | -0.53 | 8.486 | Destabilizing |
| V175E | -0.565 | 8.486 | Destabilizing |
| V175N | -0.295 | 8.486 | Destabilizing |
| V175Q | -0.296 | 8.486 | Destabilizing |
| V175H | -0.431 | 8.486 | Destabilizing |
| V175K | -0.333 | 8.486 | Destabilizing |
| V175R | -0.443 | 8.486 | Destabilizing |
| V176G | -0.218 | 4.382 | Destabilizing |
| V176A | 0.01 | 4.382 | Stabilizing |
| V176T | -0.305 | 4.382 | Destabilizing |
| V176S | -0.343 | 4.382 | Destabilizing |
| V176C | -0.353 | 4.382 | Destabilizing |
| V176L | 0.248 | 4.382 | Stabilizing |
| V176I | 0.217 | 4.382 | Stabilizing |
| V176M | -0.022 | 4.382 | Destabilizing |
| V176P | 0.182 | 4.382 | Stabilizing |
| V176F | 0.005 | 4.382 | Stabilizing |
| V176Y | -0.106 | 4.382 | Destabilizing |
| V176W | -0.355 | 4.382 | Destabilizing |
| V176D | -0.738 | 4.382 | Destabilizing |
| V176E | -0.751 | 4.382 | Destabilizing |
| V176N | -0.491 | 4.382 | Destabilizing |
| V176Q | -0.41 | 4.382 | Destabilizing |
| V176H | -0.373 | 4.382 | Destabilizing |
| V176K | -0.385 | 4.382 | Destabilizing |
| V176R | -0.535 | 4.382 | Destabilizing |
| S177G | 0.324 | 6.663 | Stabilizing |
| S177A | 0.373 | 6.663 | Stabilizing |
| S177T | 0.302 | 6.663 | Stabilizing |
| S177C | -0.071 | 6.663 | Destabilizing |
| S177V | 0.332 | 6.663 | Stabilizing |
| S177L | 0.285 | 6.663 | Stabilizing |
| S177I | 0.302 | 6.663 | Stabilizing |
| S177M | -0.005 | 6.663 | Destabilizing |
| S177P | 0.34 | 6.663 | Stabilizing |
| S177F | 0.064 | 6.663 | Stabilizing |
| S177Y | 0.057 | 6.663 | Stabilizing |
| S177W | -0.222 | 6.663 | Destabilizing |
| S177D | -0.307 | 6.663 | Destabilizing |
| S177E | -0.326 | 6.663 | Destabilizing |
| S177N | 0.151 | 6.663 | Stabilizing |
| S177Q | 0.172 | 6.663 | Stabilizing |
| S177H | -0.034 | 6.663 | Destabilizing |
| S177K | 0.12 | 6.663 | Stabilizing |
| S177R | -0.091 | 6.663 | Destabilizing |
| Q178G | 0.515 | 6.638 | Stabilizing |
| Q178A | 0.632 | 6.638 | Stabilizing |
| Q178T | 0.526 | 6.638 | Stabilizing |
| Q178S | 0.466 | 6.638 | Stabilizing |
| Q178C | 0.125 | 6.638 | Stabilizing |
| Q178V | 0.638 | 6.638 | Stabilizing |
| Q178L | 0.609 | 6.638 | Stabilizing |
| Q178I | 0.585 | 6.638 | Stabilizing |
| Q178M | 0.244 | 6.638 | Stabilizing |
| Q178P | 0.596 | 6.638 | Stabilizing |
| Q178F | 0.41 | 6.638 | Stabilizing |
| Q178Y | 0.409 | 6.638 | Stabilizing |
| Q178W | 0.131 | 6.638 | Stabilizing |
| Q178D | -0.082 | 6.638 | Destabilizing |
| Q178E | -0.021 | 6.638 | Destabilizing |
| Q178N | 0.51 | 6.638 | Stabilizing |
| Q178H | 0.185 | 6.638 | Stabilizing |
| Q178K | 0.343 | 6.638 | Stabilizing |
| Q178R | 0.038 | 6.638 | Stabilizing |
| L179G | 0.02 | 8.078 | Stabilizing |
| L179A | 0.119 | 8.078 | Stabilizing |
| L179T | -0.089 | 8.078 | Destabilizing |
| L179S | -0.1 | 8.078 | Destabilizing |
| L179C | -0.351 | 8.078 | Destabilizing |
| L179V | 0.172 | 8.078 | Stabilizing |
| L179I | 0.145 | 8.078 | Stabilizing |
| L179M | -0.219 | 8.078 | Destabilizing |
| L179P | 0.102 | 8.078 | Stabilizing |
| L179F | -0.162 | 8.078 | Destabilizing |
| L179Y | -0.204 | 8.078 | Destabilizing |
| L179W | -0.4 | 8.078 | Destabilizing |
| L179D | -0.495 | 8.078 | Destabilizing |
| L179E | -0.508 | 8.078 | Destabilizing |
| L179N | -0.198 | 8.078 | Destabilizing |
| L179Q | -0.152 | 8.078 | Destabilizing |
| L179H | -0.294 | 8.078 | Destabilizing |
| L179K | -0.189 | 8.078 | Destabilizing |
| L179R | -0.378 | 8.078 | Destabilizing |
| L384G | -0.411 | 8.669 | Destabilizing |
| L384A | -0.329 | 8.669 | Destabilizing |
| L384T | -0.502 | 8.669 | Destabilizing |
| L384S | -0.475 | 8.669 | Destabilizing |
| L384C | -0.704 | 8.669 | Destabilizing |
| L384V | -0.299 | 8.669 | Destabilizing |
| L384I | -0.306 | 8.669 | Destabilizing |
| L384M | -0.587 | 8.669 | Destabilizing |
| L384P | -0.336 | 8.669 | Destabilizing |
| L384F | -0.643 | 8.669 | Destabilizing |
| L384Y | -0.696 | 8.669 | Destabilizing |
| L384W | -0.86 | 8.669 | Destabilizing |
| L384D | -0.744 | 8.669 | Destabilizing |
| L384E | -0.78 | 8.669 | Destabilizing |
| L384N | -0.57 | 8.669 | Destabilizing |
| L384Q | -0.568 | 8.669 | Destabilizing |
| L384H | -0.8 | 8.669 | Destabilizing |
| L384K | -0.708 | 8.669 | Destabilizing |
| L384R | -0.935 | 8.669 | Destabilizing |
| E429G | -0.589 | 9.883 | Destabilizing |
| E429A | -0.594 | 9.883 | Destabilizing |
| E429T | -0.611 | 9.883 | Destabilizing |
| E429S | -0.596 | 9.883 | Destabilizing |
| E429C | -0.656 | 9.883 | Destabilizing |
| E429V | -0.609 | 9.883 | Destabilizing |
| E429L | -0.616 | 9.883 | Destabilizing |
| E429I | -0.616 | 9.883 | Destabilizing |
| E429M | -0.682 | 9.883 | Destabilizing |
| E429P | -0.608 | 9.883 | Destabilizing |
| E429F | -0.639 | 9.883 | Destabilizing |
| E429Y | -0.64 | 9.883 | Destabilizing |
| E429W | -0.658 | 9.883 | Destabilizing |
| E429D | -0.273 | 9.883 | Destabilizing |
| E429N | -0.523 | 9.883 | Destabilizing |
| E429Q | -0.497 | 9.883 | Destabilizing |
| E429H | -0.711 | 9.883 | Destabilizing |
| E429K | -0.71 | 9.883 | Destabilizing |
| E429R | -0.848 | 9.883 | Destabilizing |
| F430G | -0.636 | 7.365 | Destabilizing |
| F430A | -0.611 | 7.365 | Destabilizing |
| F430T | -0.665 | 7.365 | Destabilizing |
| F430S | -0.648 | 7.365 | Destabilizing |
| F430C | -0.797 | 7.365 | Destabilizing |
| F430V | -0.567 | 7.365 | Destabilizing |
| F430L | -0.553 | 7.365 | Destabilizing |
| F430I | -0.552 | 7.365 | Destabilizing |
| F430M | -0.754 | 7.365 | Destabilizing |
| F430P | -0.567 | 7.365 | Destabilizing |
| F430Y | -0.815 | 7.365 | Destabilizing |
| F430W | -0.992 | 7.365 | Destabilizing |
| F430D | -0.842 | 7.365 | Destabilizing |
| F430E | -0.856 | 7.365 | Destabilizing |
| F430N | -0.692 | 7.365 | Destabilizing |
| F430Q | -0.693 | 7.365 | Destabilizing |
| F430H | -0.988 | 7.365 | Destabilizing |
| F430K | -0.833 | 7.365 | Destabilizing |
| F430R | -1.01 | 7.365 | Destabilizing |
| F431G | -0.346 | 7.997 | Destabilizing |
| F431A | -0.274 | 7.997 | Destabilizing |
| F431T | -0.408 | 7.997 | Destabilizing |
| F431S | -0.409 | 7.997 | Destabilizing |
| F431C | -0.608 | 7.997 | Destabilizing |
| F431V | -0.24 | 7.997 | Destabilizing |
| F431L | -0.244 | 7.997 | Destabilizing |
| F431I | -0.245 | 7.997 | Destabilizing |
| F431M | -0.503 | 7.997 | Destabilizing |
| F431P | -0.248 | 7.997 | Destabilizing |
| F431Y | -0.509 | 7.997 | Destabilizing |
| F431W | -0.695 | 7.997 | Destabilizing |
| F431D | -0.682 | 7.997 | Destabilizing |
| F431E | -0.699 | 7.997 | Destabilizing |
| F431N | -0.447 | 7.997 | Destabilizing |
| F431Q | -0.447 | 7.997 | Destabilizing |
| F431H | -0.697 | 7.997 | Destabilizing |
| F431K | -0.553 | 7.997 | Destabilizing |
| F431R | -0.728 | 7.997 | Destabilizing |
| G432A | -0.453 | 6.857 | Destabilizing |
| G432T | -0.851 | 6.857 | Destabilizing |
| G432S | -0.837 | 6.857 | Destabilizing |
| G432C | -0.865 | 6.857 | Destabilizing |
| G432V | -0.485 | 6.857 | Destabilizing |
| G432L | -0.504 | 6.857 | Destabilizing |
| G432I | -0.534 | 6.857 | Destabilizing |
| G432M | -0.62 | 6.857 | Destabilizing |
| G432P | -0.498 | 6.857 | Destabilizing |
| G432F | -0.692 | 6.857 | Destabilizing |
| G432Y | -0.699 | 6.857 | Destabilizing |
| G432W | -0.777 | 6.857 | Destabilizing |
| G432D | -0.826 | 6.857 | Destabilizing |
| G432E | -0.83 | 6.857 | Destabilizing |
| G432N | -0.855 | 6.857 | Destabilizing |
| G432Q | -0.843 | 6.857 | Destabilizing |
| G432H | -0.791 | 6.857 | Destabilizing |
| G432K | -0.933 | 6.857 | Destabilizing |
| G432R | -0.978 | 6.857 | Destabilizing |
| T433G | -0.521 | 5.66 | Destabilizing |
| T433A | -0.556 | 5.66 | Destabilizing |
| T433S | -0.39 | 5.66 | Destabilizing |
| T433C | -0.618 | 5.66 | Destabilizing |
| T433V | -0.563 | 5.66 | Destabilizing |
| T433L | -0.576 | 5.66 | Destabilizing |
| T433I | -0.576 | 5.66 | Destabilizing |
| T433M | -0.709 | 5.66 | Destabilizing |
| T433P | -0.568 | 5.66 | Destabilizing |
| T433F | -0.656 | 5.66 | Destabilizing |
| T433Y | -0.61 | 5.66 | Destabilizing |
| T433W | -0.669 | 5.66 | Destabilizing |
| T433D | -0.477 | 5.66 | Destabilizing |
| T433E | -0.487 | 5.66 | Destabilizing |
| T433N | -0.345 | 5.66 | Destabilizing |
| T433Q | -0.373 | 5.66 | Destabilizing |
| T433H | -0.572 | 5.66 | Destabilizing |
| T433K | -0.469 | 5.66 | Destabilizing |
| T433R | -0.513 | 5.66 | Destabilizing |
| S434G | -0.624 | 3.907 | Destabilizing |
| S434A | -0.683 | 3.907 | Destabilizing |
| S434T | -0.586 | 3.907 | Destabilizing |
| S434C | -0.684 | 3.907 | Destabilizing |
| S434V | -0.668 | 3.907 | Destabilizing |
| S434L | -0.67 | 3.907 | Destabilizing |
| S434I | -0.656 | 3.907 | Destabilizing |
| S434M | -0.758 | 3.907 | Destabilizing |
| S434P | -0.656 | 3.907 | Destabilizing |
| S434F | -0.661 | 3.907 | Destabilizing |
| S434Y | -0.674 | 3.907 | Destabilizing |
| S434W | -0.653 | 3.907 | Destabilizing |
| S434D | -0.299 | 3.907 | Destabilizing |
| S434E | -0.306 | 3.907 | Destabilizing |
| S434N | -0.616 | 3.907 | Destabilizing |
| S434Q | -0.583 | 3.907 | Destabilizing |
| S434H | -0.744 | 3.907 | Destabilizing |
| S434K | -0.66 | 3.907 | Destabilizing |
| S434R | -0.868 | 3.907 | Destabilizing |
| Q435G | -1.31 | 3.328 | Destabilizing |
| Q435A | -1.302 | 3.328 | Destabilizing |
| Q435T | -1.217 | 3.328 | Destabilizing |
| Q435S | -1.23 | 3.328 | Destabilizing |
| Q435C | -1.105 | 3.328 | Destabilizing |
| Q435V | -1.234 | 3.328 | Destabilizing |
| Q435L | -1.206 | 3.328 | Destabilizing |
| Q435I | -1.207 | 3.328 | Destabilizing |
| Q435M | -1.126 | 3.328 | Destabilizing |
| Q435P | -1.224 | 3.328 | Destabilizing |
| Q435F | -0.937 | 3.328 | Destabilizing |
| Q435Y | -0.954 | 3.328 | Destabilizing |
| Q435W | -0.811 | 3.328 | Destabilizing |
| Q435D | -0.743 | 3.328 | Destabilizing |
| Q435E | -0.735 | 3.328 | Destabilizing |
| Q435N | -1.155 | 3.328 | Destabilizing |
| Q435H | -1.109 | 3.328 | Destabilizing |
| Q435K | -1.208 | 3.328 | Destabilizing |
| Q435R | -1.153 | 3.328 | Destabilizing |
| L436G | -1.144 | 3.432 | Destabilizing |
| L436A | -0.967 | 3.432 | Destabilizing |
| L436T | -1.126 | 3.432 | Destabilizing |
| L436S | -1.187 | 3.432 | Destabilizing |
| L436C | -0.895 | 3.432 | Destabilizing |
| L436V | -0.704 | 3.432 | Destabilizing |
| L436I | -0.533 | 3.432 | Destabilizing |
| L436M | -0.554 | 3.432 | Destabilizing |
| L436P | -0.747 | 3.432 | Destabilizing |
| L436F | -0.431 | 3.432 | Destabilizing |
| L436Y | -0.536 | 3.432 | Destabilizing |
| L436W | -0.489 | 3.432 | Destabilizing |
| L436D | -1.076 | 3.432 | Destabilizing |
| L436E | -1.037 | 3.432 | Destabilizing |
| L436N | -1.162 | 3.432 | Destabilizing |
| L436Q | -1.106 | 3.432 | Destabilizing |
| L436H | -0.848 | 3.432 | Destabilizing |
| L436K | -1.058 | 3.432 | Destabilizing |
| L436R | -1.055 | 3.432 | Destabilizing |
| S437G | -0.617 | 4.038 | Destabilizing |
| S437A | -0.551 | 4.038 | Destabilizing |
| S437T | -0.608 | 4.038 | Destabilizing |
| S437C | -0.581 | 4.038 | Destabilizing |
| S437V | -0.501 | 4.038 | Destabilizing |
| S437L | -0.484 | 4.038 | Destabilizing |
| S437I | -0.492 | 4.038 | Destabilizing |
| S437M | -0.528 | 4.038 | Destabilizing |
| S437P | -0.493 | 4.038 | Destabilizing |
| S437F | -0.573 | 4.038 | Destabilizing |
| S437Y | -0.615 | 4.038 | Destabilizing |
| S437W | -0.693 | 4.038 | Destabilizing |
| S437D | -0.704 | 4.038 | Destabilizing |
| S437E | -0.711 | 4.038 | Destabilizing |
| S437N | -0.816 | 4.038 | Destabilizing |
| S437Q | -0.689 | 4.038 | Destabilizing |
| S437H | -0.826 | 4.038 | Destabilizing |
| S437K | -0.725 | 4.038 | Destabilizing |
| S437R | -1.062 | 4.038 | Destabilizing |
| Q438G | -1.166 | 3.148 | Destabilizing |
| Q438A | -1.063 | 3.148 | Destabilizing |
| Q438T | -1.208 | 3.148 | Destabilizing |
| Q438S | -1.303 | 3.148 | Destabilizing |
| Q438C | -0.985 | 3.148 | Destabilizing |
| Q438V | -0.942 | 3.148 | Destabilizing |
| Q438L | -0.885 | 3.148 | Destabilizing |
| Q438I | -0.857 | 3.148 | Destabilizing |
| Q438M | -0.827 | 3.148 | Destabilizing |
| Q438P | -1.008 | 3.148 | Destabilizing |
| Q438F | -0.728 | 3.148 | Destabilizing |
| Q438Y | -0.777 | 3.148 | Destabilizing |
| Q438W | -0.686 | 3.148 | Destabilizing |
| Q438D | -1.114 | 3.148 | Destabilizing |
| Q438E | -1.076 | 3.148 | Destabilizing |
| Q438N | -1.297 | 3.148 | Destabilizing |
| Q438H | -0.936 | 3.148 | Destabilizing |
| Q438K | -1.173 | 3.148 | Destabilizing |
| Q438R | -1.106 | 3.148 | Destabilizing |
| F439G | -0.485 | 3.048 | Destabilizing |
| F439A | -0.492 | 3.048 | Destabilizing |
| F439T | -0.681 | 3.048 | Destabilizing |
| F439S | -0.669 | 3.048 | Destabilizing |
| F439C | -0.752 | 3.048 | Destabilizing |
| F439V | -0.51 | 3.048 | Destabilizing |
| F439L | -0.551 | 3.048 | Destabilizing |
| F439I | -0.51 | 3.048 | Destabilizing |
| F439M | -0.658 | 3.048 | Destabilizing |
| F439P | -0.518 | 3.048 | Destabilizing |
| F439Y | -0.759 | 3.048 | Destabilizing |
| F439W | -0.928 | 3.048 | Destabilizing |
| F439D | -0.73 | 3.048 | Destabilizing |
| F439E | -0.74 | 3.048 | Destabilizing |
| F439N | -0.767 | 3.048 | Destabilizing |
| F439Q | -0.724 | 3.048 | Destabilizing |
| F439H | -0.839 | 3.048 | Destabilizing |
| F439K | -0.783 | 3.048 | Destabilizing |
| F439R | -0.931 | 3.048 | Destabilizing |
| M440G | 0.076 | 4.525 | Stabilizing |
| M440A | 0.165 | 4.525 | Stabilizing |
| M440T | -0.091 | 4.525 | Destabilizing |
| M440S | -0.1 | 4.525 | Destabilizing |
| M440C | 0.146 | 4.525 | Stabilizing |
| M440V | 0.238 | 4.525 | Stabilizing |
| M440L | 0.29 | 4.525 | Stabilizing |
| M440I | 0.278 | 4.525 | Stabilizing |
| M440P | 0.242 | 4.525 | Stabilizing |
| M440F | 0.117 | 4.525 | Stabilizing |
| M440Y | 0.034 | 4.525 | Stabilizing |
| M440W | -0.097 | 4.525 | Destabilizing |
| M440D | -0.421 | 4.525 | Destabilizing |
| M440E | -0.433 | 4.525 | Destabilizing |
| M440N | -0.165 | 4.525 | Destabilizing |
| M440Q | -0.124 | 4.525 | Destabilizing |
| M440H | -0.117 | 4.525 | Destabilizing |
| M440K | -0.125 | 4.525 | Destabilizing |
| M440R | -0.305 | 4.525 | Destabilizing |
| D441G | -0.149 | 3.435 | Destabilizing |
| D441A | -0.18 | 3.435 | Destabilizing |
| D441C | -0.236 | 3.435 | Destabilizing |
| D441V | -0.203 | 3.435 | Destabilizing |
| D441L | -0.234 | 3.435 | Destabilizing |
| D441I | -0.22 | 3.435 | Destabilizing |
| D441M | -0.323 | 3.435 | Destabilizing |
| D441P | -0.201 | 3.435 | Destabilizing |
| D441F | -0.138 | 3.435 | Destabilizing |
| D441Y | -0.145 | 3.435 | Destabilizing |
| D441W | -0.222 | 3.435 | Destabilizing |
| D441E | 0.251 | 3.435 | Stabilizing |
| D441N | -0.108 | 3.435 | Destabilizing |
| D441Q | -0.046 | 3.435 | Destabilizing |
| D441H | -0.259 | 3.435 | Destabilizing |
| D441K | -0.173 | 3.435 | Destabilizing |
| D441R | -0.351 | 3.435 | Destabilizing |
| Q442G | 0.067 | 6.118 | Stabilizing |
| Q442A | 0.042 | 6.118 | Stabilizing |
| Q442T | 0.007 | 6.118 | Stabilizing |
| Q442S | 0.032 | 6.118 | Stabilizing |
| Q442C | -0.239 | 6.118 | Destabilizing |
| Q442V | -0.017 | 6.118 | Destabilizing |
| Q442L | -0.063 | 6.118 | Destabilizing |
| Q442I | -0.081 | 6.118 | Destabilizing |
| Q442M | -0.223 | 6.118 | Destabilizing |
| Q442P | -0.011 | 6.118 | Destabilizing |
| Q442F | 0.017 | 6.118 | Stabilizing |
| Q442W | -0.079 | 6.118 | Destabilizing |
| Q442D | -0.193 | 6.118 | Destabilizing |
| Q442E | -0.185 | 6.118 | Destabilizing |
| Q442N | 0.112 | 6.118 | Stabilizing |
| Q442H | -0.125 | 6.118 | Destabilizing |
| Q442K | -0.126 | 6.118 | Destabilizing |
| Q442R | -0.312 | 6.118 | Destabilizing |
| N443G | 0.032 | 6.377 | Stabilizing |
| N443A | 0.007 | 6.377 | Stabilizing |
| N443T | 0.037 | 6.377 | Stabilizing |
| N443S | 0.068 | 6.377 | Stabilizing |
| N443C | -0.192 | 6.377 | Destabilizing |
| N443V | -0.043 | 6.377 | Destabilizing |
| N443L | -0.078 | 6.377 | Destabilizing |
| N443I | -0.085 | 6.377 | Destabilizing |
| N443M | -0.211 | 6.377 | Destabilizing |
| N443P | -0.038 | 6.377 | Destabilizing |
| N443F | -0.053 | 6.377 | Destabilizing |
| N443Y | -0.044 | 6.377 | Destabilizing |
| N443W | -0.147 | 6.377 | Destabilizing |
| N443D | -0.146 | 6.377 | Destabilizing |
| N443E | -0.143 | 6.377 | Destabilizing |
| N443Q | 0.164 | 6.377 | Stabilizing |
| N443H | -0.118 | 6.377 | Destabilizing |
| N443K | -0.079 | 6.377 | Destabilizing |
| N443R | -0.184 | 6.377 | Destabilizing |
| S447G | 0.332 | 7.16 | Stabilizing |
| S447A | 0.329 | 7.16 | Stabilizing |
| S447T | 0.369 | 7.16 | Stabilizing |
| S447C | 0.067 | 7.16 | Stabilizing |
| S447V | 0.279 | 7.16 | Stabilizing |
| S447L | 0.249 | 7.16 | Stabilizing |
| S447I | 0.249 | 7.16 | Stabilizing |
| S447M | 0.043 | 7.16 | Stabilizing |
| S447P | 0.283 | 7.16 | Stabilizing |
| S447F | 0.142 | 7.16 | Stabilizing |
| S447Y | 0.131 | 7.16 | Stabilizing |
| S447W | -0.08 | 7.16 | Destabilizing |
| S447D | -0.226 | 7.16 | Destabilizing |
| S447E | -0.245 | 7.16 | Destabilizing |
| S447N | 0.281 | 7.16 | Stabilizing |
| S447Q | 0.285 | 7.16 | Stabilizing |
| S447H | 0.015 | 7.16 | Stabilizing |
| S447K | 0.188 | 7.16 | Stabilizing |
| S447R | -0.035 | 7.16 | Destabilizing |
| G448A | 0.243 | 8.941 | Stabilizing |
| G448T | 0.014 | 8.941 | Stabilizing |
| G448S | 0.053 | 8.941 | Stabilizing |
| G448C | -0.221 | 8.941 | Destabilizing |
| G448V | 0.138 | 8.941 | Stabilizing |
| G448L | 0.072 | 8.941 | Stabilizing |
| G448I | 0.073 | 8.941 | Stabilizing |
| G448M | -0.112 | 8.941 | Destabilizing |
| G448P | 0.144 | 8.941 | Stabilizing |
| G448F | -0.115 | 8.941 | Destabilizing |
| G448Y | -0.118 | 8.941 | Destabilizing |
| G448W | -0.308 | 8.941 | Destabilizing |
| G448D | -0.378 | 8.941 | Destabilizing |
| G448E | -0.394 | 8.941 | Destabilizing |
| G448N | -0.07 | 8.941 | Destabilizing |
| G448Q | -0.062 | 8.941 | Destabilizing |
| G448H | -0.151 | 8.941 | Destabilizing |
| G448K | -0.072 | 8.941 | Destabilizing |
| G448R | -0.254 | 8.941 | Destabilizing |
| T450G | 0.547 | 8.478 | Stabilizing |
| T450A | 0.672 | 8.478 | Stabilizing |
| T450S | 0.592 | 8.478 | Stabilizing |
| T450C | 0.152 | 8.478 | Stabilizing |
| T450V | 0.667 | 8.478 | Stabilizing |
| T450L | 0.631 | 8.478 | Stabilizing |
| T450I | 0.626 | 8.478 | Stabilizing |
| T450M | 0.281 | 8.478 | Stabilizing |
| T450P | 0.666 | 8.478 | Stabilizing |
| T450F | 0.407 | 8.478 | Stabilizing |
| T450Y | 0.41 | 8.478 | Stabilizing |
| T450W | 0.145 | 8.478 | Stabilizing |
| T450D | -0.112 | 8.478 | Destabilizing |
| T450E | -0.125 | 8.478 | Destabilizing |
| T450N | 0.44 | 8.478 | Stabilizing |
| T450Q | 0.445 | 8.478 | Stabilizing |
| T450H | 0.211 | 8.478 | Stabilizing |
| T450K | 0.443 | 8.478 | Stabilizing |
| T450R | 0.152 | 8.478 | Stabilizing |
| H451G | -0.709 | 3.714 | Destabilizing |
| H451A | -0.561 | 3.714 | Destabilizing |
| H451T | -0.677 | 3.714 | Destabilizing |
| H451S | -0.737 | 3.714 | Destabilizing |
| H451C | -0.621 | 3.714 | Destabilizing |
| H451V | -0.382 | 3.714 | Destabilizing |
| H451L | -0.312 | 3.714 | Destabilizing |
| H451I | -0.313 | 3.714 | Destabilizing |
| H451M | -0.411 | 3.714 | Destabilizing |
| H451P | -0.48 | 3.714 | Destabilizing |
| H451F | -0.124 | 3.714 | Destabilizing |
| H451Y | -0.091 | 3.714 | Destabilizing |
| H451W | -0.076 | 3.714 | Destabilizing |
| H451D | -0.756 | 3.714 | Destabilizing |
| H451E | -0.777 | 3.714 | Destabilizing |
| H451N | -0.747 | 3.714 | Destabilizing |
| H451Q | -0.682 | 3.714 | Destabilizing |
| H451K | -0.653 | 3.714 | Destabilizing |
| H451R | -0.584 | 3.714 | Destabilizing |
| K452G | 0.18 | 8.696 | Stabilizing |
| K452A | 0.222 | 8.696 | Stabilizing |
| K452T | 0.222 | 8.696 | Stabilizing |
| K452S | 0.209 | 8.696 | Stabilizing |
| K452C | -0.11 | 8.696 | Destabilizing |
| K452V | 0.188 | 8.696 | Stabilizing |
| K452L | 0.132 | 8.696 | Stabilizing |
| K452I | 0.137 | 8.696 | Stabilizing |
| K452M | -0.083 | 8.696 | Destabilizing |
| K452P | 0.19 | 8.696 | Stabilizing |
| K452F | 0.018 | 8.696 | Stabilizing |
| K452Y | 0.015 | 8.696 | Stabilizing |
| K452W | -0.14 | 8.696 | Destabilizing |
| K452D | -0.252 | 8.696 | Destabilizing |
| K452E | -0.252 | 8.696 | Destabilizing |
| K452N | 0.15 | 8.696 | Stabilizing |
| K452Q | 0.168 | 8.696 | Stabilizing |
| K452H | 0.082 | 8.696 | Stabilizing |
| K452R | 0.125 | 8.696 | Stabilizing |
| R453G | -0.181 | 9.155 | Destabilizing |
| R453A | -0.097 | 9.155 | Destabilizing |
| R453T | -0.02 | 9.155 | Destabilizing |
| R453S | -0.041 | 9.155 | Destabilizing |
| R453C | -0.342 | 9.155 | Destabilizing |
| R453V | -0.046 | 9.155 | Destabilizing |
| R453L | -0.038 | 9.155 | Destabilizing |
| R453I | -0.038 | 9.155 | Destabilizing |
| R453M | -0.271 | 9.155 | Destabilizing |
| R453P | -0.076 | 9.155 | Destabilizing |
| R453F | -0.188 | 9.155 | Destabilizing |
| R453Y | -0.116 | 9.155 | Destabilizing |
| R453W | -0.256 | 9.155 | Destabilizing |
| R453D | -0.417 | 9.155 | Destabilizing |
| R453E | -0.433 | 9.155 | Destabilizing |
| R453N | -0.029 | 9.155 | Destabilizing |
| R453Q | -0.056 | 9.155 | Destabilizing |
| R453H | 0.085 | 9.155 | Stabilizing |
| R453K | 0.029 | 9.155 | Stabilizing |
| R454G | -0.81 | 2.443 | Destabilizing |
| R454A | -0.704 | 2.443 | Destabilizing |
| R454T | -0.775 | 2.443 | Destabilizing |
| R454S | -0.889 | 2.443 | Destabilizing |
| R454C | -0.719 | 2.443 | Destabilizing |
| R454V | -0.508 | 2.443 | Destabilizing |
| R454L | -0.453 | 2.443 | Destabilizing |
| R454I | -0.458 | 2.443 | Destabilizing |
| R454M | -0.54 | 2.443 | Destabilizing |
| R454P | -0.534 | 2.443 | Destabilizing |
| R454F | -0.33 | 2.443 | Destabilizing |
| R454Y | -0.28 | 2.443 | Destabilizing |
| R454W | -0.262 | 2.443 | Destabilizing |
| R454D | -0.723 | 2.443 | Destabilizing |
| R454E | -0.684 | 2.443 | Destabilizing |
| R454N | -0.793 | 2.443 | Destabilizing |
| R454Q | -0.717 | 2.443 | Destabilizing |
| R454H | -0.501 | 2.443 | Destabilizing |
| R454K | -0.754 | 2.443 | Destabilizing |
| L455G | -0.073 | 5.924 | Destabilizing |
| L455A | 0.071 | 5.924 | Stabilizing |
| L455T | -0.184 | 5.924 | Destabilizing |
| L455S | -0.206 | 5.924 | Destabilizing |
| L455C | -0.373 | 5.924 | Destabilizing |
| L455V | 0.207 | 5.924 | Stabilizing |
| L455I | 0.221 | 5.924 | Stabilizing |
| L455M | -0.149 | 5.924 | Destabilizing |
| L455P | 0.017 | 5.924 | Stabilizing |
| L455F | -0.081 | 5.924 | Destabilizing |
| L455Y | -0.157 | 5.924 | Destabilizing |
| L455W | -0.341 | 5.924 | Destabilizing |
| L455D | -0.582 | 5.924 | Destabilizing |
| L455E | -0.598 | 5.924 | Destabilizing |
| L455N | -0.278 | 5.924 | Destabilizing |
| L455Q | -0.246 | 5.924 | Destabilizing |
| L455H | -0.332 | 5.924 | Destabilizing |
| L455K | -0.266 | 5.924 | Destabilizing |
| L455R | -0.487 | 5.924 | Destabilizing |
| S456G | -0.573 | 2.66 | Destabilizing |
| S456A | -0.392 | 2.66 | Destabilizing |
| S456T | -0.51 | 2.66 | Destabilizing |
| S456C | -0.44 | 2.66 | Destabilizing |
| S456V | -0.314 | 2.66 | Destabilizing |
| S456L | -0.303 | 2.66 | Destabilizing |
| S456I | -0.295 | 2.66 | Destabilizing |
| S456M | -0.365 | 2.66 | Destabilizing |
| S456P | -0.446 | 2.66 | Destabilizing |
| S456F | -0.279 | 2.66 | Destabilizing |
| S456Y | -0.355 | 2.66 | Destabilizing |
| S456W | -0.45 | 2.66 | Destabilizing |
| S456D | -0.705 | 2.66 | Destabilizing |
| S456E | -0.732 | 2.66 | Destabilizing |
| S456N | -0.671 | 2.66 | Destabilizing |
| S456Q | -0.597 | 2.66 | Destabilizing |
| S456H | -0.637 | 2.66 | Destabilizing |
| S456K | -0.644 | 2.66 | Destabilizing |
| S456R | -0.817 | 2.66 | Destabilizing |
| A457G | 0.039 | 5.726 | Stabilizing |
| A457T | -0.381 | 5.726 | Destabilizing |
| A457S | -0.384 | 5.726 | Destabilizing |
| A457C | -0.444 | 5.726 | Destabilizing |
| A457V | 0.164 | 5.726 | Stabilizing |
| A457L | 0.157 | 5.726 | Stabilizing |
| A457I | 0.155 | 5.726 | Stabilizing |
| A457M | -0.102 | 5.726 | Destabilizing |
| A457P | 0.119 | 5.726 | Stabilizing |
| A457F | -0.135 | 5.726 | Destabilizing |
| A457Y | -0.255 | 5.726 | Destabilizing |
| A457W | -0.5 | 5.726 | Destabilizing |
| A457D | -0.82 | 5.726 | Destabilizing |
| A457E | -0.843 | 5.726 | Destabilizing |
| A457N | -0.612 | 5.726 | Destabilizing |
| A457Q | -0.534 | 5.726 | Destabilizing |
| A457H | -0.556 | 5.726 | Destabilizing |
| A457K | -0.566 | 5.726 | Destabilizing |
| A457R | -0.845 | 5.726 | Destabilizing |
| L458G | -1.519 | 3.417 | Destabilizing |
| L458A | -1.425 | 3.417 | Destabilizing |
| L458T | -1.383 | 3.417 | Destabilizing |
| L458S | -1.41 | 3.417 | Destabilizing |
| L458C | -1.164 | 3.417 | Destabilizing |
| L458V | -1.264 | 3.417 | Destabilizing |
| L458I | -1.145 | 3.417 | Destabilizing |
| L458M | -1.064 | 3.417 | Destabilizing |
| L458P | -1.274 | 3.417 | Destabilizing |
| L458F | -0.905 | 3.417 | Destabilizing |
| L458Y | -0.934 | 3.417 | Destabilizing |
| L458W | -0.793 | 3.417 | Destabilizing |
| L458D | -1.167 | 3.417 | Destabilizing |
| L458E | -1.144 | 3.417 | Destabilizing |
| L458N | -1.319 | 3.417 | Destabilizing |
| L458Q | -1.315 | 3.417 | Destabilizing |
| L458H | -1.136 | 3.417 | Destabilizing |
| L458K | -1.308 | 3.417 | Destabilizing |
| L458R | -1.175 | 3.417 | Destabilizing |
| G459A | -0.715 | 4.641 | Destabilizing |
| G459T | -0.998 | 4.641 | Destabilizing |
| G459S | -0.944 | 4.641 | Destabilizing |
| G459C | -1.002 | 4.641 | Destabilizing |
| G459V | -0.837 | 4.641 | Destabilizing |
| G459L | -0.902 | 4.641 | Destabilizing |
| G459I | -0.799 | 4.641 | Destabilizing |
| G459M | -0.959 | 4.641 | Destabilizing |
| G459P | -0.73 | 4.641 | Destabilizing |
| G459F | -1.102 | 4.641 | Destabilizing |
| G459Y | -1.102 | 4.641 | Destabilizing |
| G459W | -1.019 | 4.641 | Destabilizing |
| G459D | -0.93 | 4.641 | Destabilizing |
| G459E | -0.958 | 4.641 | Destabilizing |
| G459N | -1.068 | 4.641 | Destabilizing |
| G459Q | -1.068 | 4.641 | Destabilizing |
| G459H | -1.172 | 4.641 | Destabilizing |
| G459K | -1.095 | 4.641 | Destabilizing |
| G459R | -1.236 | 4.641 | Destabilizing |
| P460G | -0.275 | 7.03 | Destabilizing |
| P460A | -0.348 | 7.03 | Destabilizing |
| P460T | -0.709 | 7.03 | Destabilizing |
| P460S | -0.687 | 7.03 | Destabilizing |
| P460C | -0.785 | 7.03 | Destabilizing |
| P460V | -0.404 | 7.03 | Destabilizing |
| P460L | -0.441 | 7.03 | Destabilizing |
| P460I | -0.452 | 7.03 | Destabilizing |
| P460M | -0.551 | 7.03 | Destabilizing |
| P460F | -0.699 | 7.03 | Destabilizing |
| P460Y | -0.715 | 7.03 | Destabilizing |
| P460W | -0.837 | 7.03 | Destabilizing |
| P460D | -0.741 | 7.03 | Destabilizing |
| P460E | -0.762 | 7.03 | Destabilizing |
| P460N | -0.731 | 7.03 | Destabilizing |
| P460Q | -0.742 | 7.03 | Destabilizing |
| P460H | -0.786 | 7.03 | Destabilizing |
| P460K | -0.832 | 7.03 | Destabilizing |
| P460R | -0.947 | 7.03 | Destabilizing |
| G462A | -0.281 | 8.979 | Destabilizing |
| G462T | -0.77 | 8.979 | Destabilizing |
| G462S | -0.748 | 8.979 | Destabilizing |
| G462C | -0.843 | 8.979 | Destabilizing |
| G462V | -0.297 | 8.979 | Destabilizing |
| G462L | -0.308 | 8.979 | Destabilizing |
| G462I | -0.323 | 8.979 | Destabilizing |
| G462M | -0.522 | 8.979 | Destabilizing |
| G462P | -0.287 | 8.979 | Destabilizing |
| G462F | -0.515 | 8.979 | Destabilizing |
| G462Y | -0.533 | 8.979 | Destabilizing |
| G462W | -0.631 | 8.979 | Destabilizing |
| G462D | -0.768 | 8.979 | Destabilizing |
| G462E | -0.783 | 8.979 | Destabilizing |
| G462N | -0.791 | 8.979 | Destabilizing |
| G462Q | -0.761 | 8.979 | Destabilizing |
| G462H | -0.712 | 8.979 | Destabilizing |
| G462K | -0.846 | 8.979 | Destabilizing |
| G462R | -0.959 | 8.979 | Destabilizing |
| L463G | -0.157 | 6.063 | Destabilizing |
| L463A | -0.099 | 6.063 | Destabilizing |
| L463T | -0.596 | 6.063 | Destabilizing |
| L463S | -0.579 | 6.063 | Destabilizing |
| L463C | -0.715 | 6.063 | Destabilizing |
| L463V | -0.036 | 6.063 | Destabilizing |
| L463I | -0.035 | 6.063 | Destabilizing |
| L463M | -0.343 | 6.063 | Destabilizing |
| L463P | -0.123 | 6.063 | Destabilizing |
| L463F | -0.276 | 6.063 | Destabilizing |
| L463Y | -0.345 | 6.063 | Destabilizing |
| L463W | -0.49 | 6.063 | Destabilizing |
| L463D | -0.751 | 6.063 | Destabilizing |
| L463E | -0.75 | 6.063 | Destabilizing |
| L463N | -0.703 | 6.063 | Destabilizing |
| L463Q | -0.647 | 6.063 | Destabilizing |
| L463H | -0.623 | 6.063 | Destabilizing |
| L463K | -0.723 | 6.063 | Destabilizing |
| L463R | -0.937 | 6.063 | Destabilizing |
| S464G | -0.424 | 6.571 | Destabilizing |
| S464A | -0.462 | 6.571 | Destabilizing |
| S464T | -0.42 | 6.571 | Destabilizing |
| S464C | -0.653 | 6.571 | Destabilizing |
| S464V | -0.476 | 6.571 | Destabilizing |
| S464L | -0.481 | 6.571 | Destabilizing |
| S464I | -0.491 | 6.571 | Destabilizing |
| S464M | -0.669 | 6.571 | Destabilizing |
| S464P | -0.495 | 6.571 | Destabilizing |
| S464F | -0.592 | 6.571 | Destabilizing |
| S464Y | -0.568 | 6.571 | Destabilizing |
| S464W | -0.639 | 6.571 | Destabilizing |
| S464D | -0.488 | 6.571 | Destabilizing |
| S464E | -0.503 | 6.571 | Destabilizing |
| S464N | -0.365 | 6.571 | Destabilizing |
| S464Q | -0.405 | 6.571 | Destabilizing |
| S464H | -0.613 | 6.571 | Destabilizing |
| S464K | -0.527 | 6.571 | Destabilizing |
| S464R | -0.659 | 6.571 | Destabilizing |
| R465G | -0.713 | 2.759 | Destabilizing |
| R465A | -0.69 | 2.759 | Destabilizing |
| R465T | -0.732 | 2.759 | Destabilizing |
| R465S | -0.734 | 2.759 | Destabilizing |
| R465C | -0.833 | 2.759 | Destabilizing |
| R465V | -0.635 | 2.759 | Destabilizing |
| R465L | -0.631 | 2.759 | Destabilizing |
| R465I | -0.622 | 2.759 | Destabilizing |
| R465M | -0.819 | 2.759 | Destabilizing |
| R465P | -0.634 | 2.759 | Destabilizing |
| R465F | -0.532 | 2.759 | Destabilizing |
| R465Y | -0.59 | 2.759 | Destabilizing |
| R465W | -0.585 | 2.759 | Destabilizing |
| R465D | -0.494 | 2.759 | Destabilizing |
| R465E | -0.488 | 2.759 | Destabilizing |
| R465N | -0.705 | 2.759 | Destabilizing |
| R465Q | -0.651 | 2.759 | Destabilizing |
| R465H | -1.186 | 2.759 | Destabilizing |
| R465K | -0.94 | 2.759 | Destabilizing |
| E466G | -0.667 | 8.541 | Destabilizing |
| E466A | -0.664 | 8.541 | Destabilizing |
| E466T | -0.652 | 8.541 | Destabilizing |
| E466S | -0.646 | 8.541 | Destabilizing |
| E466C | -0.677 | 8.541 | Destabilizing |
| E466V | -0.668 | 8.541 | Destabilizing |
| E466L | -0.671 | 8.541 | Destabilizing |
| E466I | -0.67 | 8.541 | Destabilizing |
| E466M | -0.708 | 8.541 | Destabilizing |
| E466P | -0.669 | 8.541 | Destabilizing |
| E466F | -0.67 | 8.541 | Destabilizing |
| E466Y | -0.665 | 8.541 | Destabilizing |
| E466W | -0.666 | 8.541 | Destabilizing |
| E466D | -0.402 | 8.541 | Destabilizing |
| E466N | -0.577 | 8.541 | Destabilizing |
| E466Q | -0.568 | 8.541 | Destabilizing |
| E466H | -0.698 | 8.541 | Destabilizing |
| E466K | -0.688 | 8.541 | Destabilizing |
| E466R | -0.74 | 8.541 | Destabilizing |
| I486G | 0.009 | 9.273 | Stabilizing |
| I486A | 0.077 | 9.273 | Stabilizing |
| I486T | -0.097 | 9.273 | Destabilizing |
| I486S | -0.093 | 9.273 | Destabilizing |
| I486C | -0.351 | 9.273 | Destabilizing |
| I486V | 0.086 | 9.273 | Stabilizing |
| I486L | 0.069 | 9.273 | Stabilizing |
| I486M | -0.241 | 9.273 | Destabilizing |
| I486P | 0.056 | 9.273 | Stabilizing |
| I486F | -0.167 | 9.273 | Destabilizing |
| I486Y | -0.205 | 9.273 | Destabilizing |
| I486W | -0.36 | 9.273 | Destabilizing |
| I486D | -0.437 | 9.273 | Destabilizing |
| I486E | -0.449 | 9.273 | Destabilizing |
| I486N | -0.185 | 9.273 | Destabilizing |
| I486Q | -0.167 | 9.273 | Destabilizing |
| I486H | -0.225 | 9.273 | Destabilizing |
| I486K | -0.156 | 9.273 | Destabilizing |
| I486R | -0.316 | 9.273 | Destabilizing |
| E487G | -0.099 | 7.367 | Destabilizing |
| E487A | -0.122 | 7.367 | Destabilizing |
| E487T | -0.181 | 7.367 | Destabilizing |
| E487S | -0.168 | 7.367 | Destabilizing |
| E487C | -0.294 | 7.367 | Destabilizing |
| E487V | -0.152 | 7.367 | Destabilizing |
| E487L | -0.155 | 7.367 | Destabilizing |
| E487I | -0.181 | 7.367 | Destabilizing |
| E487M | -0.276 | 7.367 | Destabilizing |
| E487P | -0.141 | 7.367 | Destabilizing |
| E487F | -0.096 | 7.367 | Destabilizing |
| E487Y | -0.125 | 7.367 | Destabilizing |
| E487W | -0.233 | 7.367 | Destabilizing |
| E487D | 0.219 | 7.367 | Stabilizing |
| E487N | -0.159 | 7.367 | Destabilizing |
| E487Q | -0.11 | 7.367 | Destabilizing |
| E487H | -0.236 | 7.367 | Destabilizing |
| E487K | -0.22 | 7.367 | Destabilizing |
| E487R | -0.348 | 7.367 | Destabilizing |
| T488G | 0.482 | 4.92 | Stabilizing |
| T488A | 0.605 | 4.92 | Stabilizing |
| T488S | 0.463 | 4.92 | Stabilizing |
| T488C | 0.069 | 4.92 | Stabilizing |
| T488V | 0.578 | 4.92 | Stabilizing |
| T488L | 0.578 | 4.92 | Stabilizing |
| T488I | 0.538 | 4.92 | Stabilizing |
| T488M | 0.239 | 4.92 | Stabilizing |
| T488P | 0.496 | 4.92 | Stabilizing |
| T488F | 0.365 | 4.92 | Stabilizing |
| T488Y | 0.383 | 4.92 | Stabilizing |
| T488W | 0.136 | 4.92 | Stabilizing |
| T488D | -0.178 | 4.92 | Destabilizing |
| T488E | -0.188 | 4.92 | Destabilizing |
| T488N | 0.321 | 4.92 | Stabilizing |
| T488Q | 0.326 | 4.92 | Stabilizing |
| T488H | 0.124 | 4.92 | Stabilizing |
| T488K | 0.32 | 4.92 | Stabilizing |
| P489G | -0.563 | 3.023 | Destabilizing |
| P489A | -0.421 | 3.023 | Destabilizing |
| P489T | -0.777 | 3.023 | Destabilizing |
| P489S | -0.791 | 3.023 | Destabilizing |
| P489C | -0.688 | 3.023 | Destabilizing |
| P489V | -0.308 | 3.023 | Destabilizing |
| P489L | -0.254 | 3.023 | Destabilizing |
| P489I | -0.269 | 3.023 | Destabilizing |
| P489M | -0.389 | 3.023 | Destabilizing |
| P489F | -0.295 | 3.023 | Destabilizing |
| P489Y | -0.351 | 3.023 | Destabilizing |
| P489W | -0.425 | 3.023 | Destabilizing |
| P489D | -0.875 | 3.023 | Destabilizing |
| P489E | -0.878 | 3.023 | Destabilizing |
| P489N | -0.881 | 3.023 | Destabilizing |
| P489Q | -0.826 | 3.023 | Destabilizing |
| P489H | -0.659 | 3.023 | Destabilizing |
| P489K | -0.796 | 3.023 | Destabilizing |
| P489R | -0.917 | 3.023 | Destabilizing |
| E490G | -0.504 | 5.45 | Destabilizing |
| E490A | -0.507 | 5.45 | Destabilizing |
| E490T | -0.671 | 5.45 | Destabilizing |
| E490S | -0.659 | 5.45 | Destabilizing |
| E490C | -0.754 | 5.45 | Destabilizing |
| E490V | -0.517 | 5.45 | Destabilizing |
| E490L | -0.526 | 5.45 | Destabilizing |
| E490I | -0.525 | 5.45 | Destabilizing |
| E490M | -0.643 | 5.45 | Destabilizing |
| E490P | -0.509 | 5.45 | Destabilizing |
| E490F | -0.542 | 5.45 | Destabilizing |
| E490Y | -0.565 | 5.45 | Destabilizing |
| E490W | -0.587 | 5.45 | Destabilizing |
| E490D | -0.331 | 5.45 | Destabilizing |
| E490N | -0.622 | 5.45 | Destabilizing |
| E490Q | -0.583 | 5.45 | Destabilizing |
| E490H | -0.655 | 5.45 | Destabilizing |
| E490K | -0.736 | 5.45 | Destabilizing |
| E490R | -0.846 | 5.45 | Destabilizing |
| G491A | -0.362 | 6.306 | Destabilizing |
| G491T | -0.704 | 6.306 | Destabilizing |
| G491S | -0.685 | 6.306 | Destabilizing |
| G491C | -0.764 | 6.306 | Destabilizing |
| G491V | -0.386 | 6.306 | Destabilizing |
| G491L | -0.481 | 6.306 | Destabilizing |
| G491I | -0.434 | 6.306 | Destabilizing |
| G491M | -0.531 | 6.306 | Destabilizing |
| G491P | -0.449 | 6.306 | Destabilizing |
| G491F | -0.528 | 6.306 | Destabilizing |
| G491Y | -0.574 | 6.306 | Destabilizing |
| G491W | -0.669 | 6.306 | Destabilizing |
| G491D | -0.625 | 6.306 | Destabilizing |
| G491E | -0.617 | 6.306 | Destabilizing |
| G491N | -0.801 | 6.306 | Destabilizing |
| G491Q | -0.74 | 6.306 | Destabilizing |
| G491H | -0.724 | 6.306 | Destabilizing |
| G491K | -0.747 | 6.306 | Destabilizing |
| G491R | -0.882 | 6.306 | Destabilizing |
| P492G | -0.604 | 4.964 | Destabilizing |
| P492A | -0.758 | 4.964 | Destabilizing |
| P492T | -0.99 | 4.964 | Destabilizing |
| P492S | -0.935 | 4.964 | Destabilizing |
| P492C | -1.014 | 4.964 | Destabilizing |
| P492V | -0.819 | 4.964 | Destabilizing |
| P492L | -0.9 | 4.964 | Destabilizing |
| P492I | -0.874 | 4.964 | Destabilizing |
| P492M | -0.928 | 4.964 | Destabilizing |
| P492F | -1.051 | 4.964 | Destabilizing |
| P492Y | -1.09 | 4.964 | Destabilizing |
| P492W | -1.135 | 4.964 | Destabilizing |
| P492D | -0.855 | 4.964 | Destabilizing |
| P492E | -0.897 | 4.964 | Destabilizing |
| P492N | -1.026 | 4.964 | Destabilizing |
| P492Q | -1.051 | 4.964 | Destabilizing |
| P492H | -1.134 | 4.964 | Destabilizing |
| P492K | -1.103 | 4.964 | Destabilizing |
| P492R | -1.16 | 4.964 | Destabilizing |
| N493G | -1.128 | 2.726 | Destabilizing |
| N493A | -1.044 | 2.726 | Destabilizing |
| N493T | -1.091 | 2.726 | Destabilizing |
| N493S | -1.165 | 2.726 | Destabilizing |
| N493C | -0.931 | 2.726 | Destabilizing |
| N493V | -0.923 | 2.726 | Destabilizing |
| N493L | -0.872 | 2.726 | Destabilizing |
| N493I | -0.87 | 2.726 | Destabilizing |
| N493M | -0.847 | 2.726 | Destabilizing |
| N493P | -0.925 | 2.726 | Destabilizing |
| N493F | -0.597 | 2.726 | Destabilizing |
| N493Y | -0.64 | 2.726 | Destabilizing |
| N493W | -0.528 | 2.726 | Destabilizing |
| N493D | -0.758 | 2.726 | Destabilizing |
| N493E | -0.738 | 2.726 | Destabilizing |
| N493Q | -1.077 | 2.726 | Destabilizing |
| N493H | -1.016 | 2.726 | Destabilizing |
| N493K | -1.109 | 2.726 | Destabilizing |
| N493R | -1.188 | 2.726 | Destabilizing |
| I494G | -0.227 | 7.636 | Destabilizing |
| I494A | -0.154 | 7.636 | Destabilizing |
| I494T | -0.494 | 7.636 | Destabilizing |
| I494S | -0.469 | 7.636 | Destabilizing |
| I494C | -0.688 | 7.636 | Destabilizing |
| I494V | -0.103 | 7.636 | Destabilizing |
| I494L | -0.11 | 7.636 | Destabilizing |
| I494M | -0.42 | 7.636 | Destabilizing |
| I494P | -0.106 | 7.636 | Destabilizing |
| I494F | -0.431 | 7.636 | Destabilizing |
| I494Y | -0.489 | 7.636 | Destabilizing |
| I494W | -0.666 | 7.636 | Destabilizing |
| I494D | -0.758 | 7.636 | Destabilizing |
| I494E | -0.765 | 7.636 | Destabilizing |
| I494N | -0.588 | 7.636 | Destabilizing |
| I494Q | -0.556 | 7.636 | Destabilizing |
| I494H | -0.715 | 7.636 | Destabilizing |
| I494K | -0.691 | 7.636 | Destabilizing |
| I494R | -0.986 | 7.636 | Destabilizing |
| G495A | 0.317 | 8.22 | Stabilizing |
| G495T | -0.226 | 8.22 | Destabilizing |
| G495S | -0.199 | 8.22 | Destabilizing |
| G495C | -0.41 | 8.22 | Destabilizing |
| G495V | 0.266 | 8.22 | Stabilizing |
| G495L | 0.233 | 8.22 | Stabilizing |
| G495I | 0.236 | 8.22 | Stabilizing |
| G495M | -0.088 | 8.22 | Destabilizing |
| G495P | 0.039 | 8.22 | Stabilizing |
| G495F | -0.084 | 8.22 | Destabilizing |
| G495Y | -0.149 | 8.22 | Destabilizing |
| G495W | -0.364 | 8.22 | Destabilizing |
| G495D | -0.57 | 8.22 | Destabilizing |
| G495E | -0.583 | 8.22 | Destabilizing |
| G495N | -0.352 | 8.22 | Destabilizing |
| G495Q | -0.305 | 8.22 | Destabilizing |
| G495H | -0.346 | 8.22 | Destabilizing |
| G495K | -0.366 | 8.22 | Destabilizing |
| G495R | -0.61 | 8.22 | Destabilizing |
| L496G | -0.454 | 6.06 | Destabilizing |
| L496A | -0.244 | 6.06 | Destabilizing |
| L496T | -0.457 | 6.06 | Destabilizing |
| L496S | -0.507 | 6.06 | Destabilizing |
| L496C | -0.447 | 6.06 | Destabilizing |
| L496V | -0.007 | 6.06 | Destabilizing |
| L496I | 0.126 | 6.06 | Stabilizing |
| L496M | -0.105 | 6.06 | Destabilizing |
| L496P | -0.055 | 6.06 | Destabilizing |
| L496Y | -0.125 | 6.06 | Destabilizing |
| L496W | -0.24 | 6.06 | Destabilizing |
| L496D | -0.824 | 6.06 | Destabilizing |
| L496E | -0.806 | 6.06 | Destabilizing |
| L496N | -0.58 | 6.06 | Destabilizing |
| L496Q | -0.516 | 6.06 | Destabilizing |
| L496H | -0.446 | 6.06 | Destabilizing |
| L496K | -0.492 | 6.06 | Destabilizing |
| L496R | -0.654 | 6.06 | Destabilizing |
| I497G | -1.151 | 2.292 | Destabilizing |
| I497A | -1.034 | 2.292 | Destabilizing |
| I497T | -1.092 | 2.292 | Destabilizing |
| I497S | -1.121 | 2.292 | Destabilizing |
| I497C | -0.884 | 2.292 | Destabilizing |
| I497L | -0.712 | 2.292 | Destabilizing |
| I497M | -0.704 | 2.292 | Destabilizing |
| I497P | -0.962 | 2.292 | Destabilizing |
| I497F | -0.529 | 2.292 | Destabilizing |
| I497D | -1.043 | 2.292 | Destabilizing |
| I497E | -1.029 | 2.292 | Destabilizing |
| I497N | -1.063 | 2.292 | Destabilizing |
| I497Q | -1.069 | 2.292 | Destabilizing |
| I497H | -0.793 | 2.292 | Destabilizing |
| I497K | -1.019 | 2.292 | Destabilizing |
| I497R | -0.934 | 2.292 | Destabilizing |
| G498A | 0.569 | 6.057 | Stabilizing |
| G498T | 0.043 | 6.057 | Stabilizing |
| G498S | 0.049 | 6.057 | Stabilizing |
| G498C | -0.143 | 6.057 | Destabilizing |
| G498V | 0.526 | 6.057 | Stabilizing |
| G498L | 0.503 | 6.057 | Stabilizing |
| G498I | 0.477 | 6.057 | Stabilizing |
| G498M | 0.218 | 6.057 | Stabilizing |
| G498P | 0.425 | 6.057 | Stabilizing |
| G498F | 0.254 | 6.057 | Stabilizing |
| G498Y | 0.189 | 6.057 | Stabilizing |
| G498W | -0.047 | 6.057 | Destabilizing |
| G498D | -0.431 | 6.057 | Destabilizing |
| G498E | -0.436 | 6.057 | Destabilizing |
| G498N | -0.132 | 6.057 | Destabilizing |
| G498Q | -0.075 | 6.057 | Destabilizing |
| G498H | -0.012 | 6.057 | Destabilizing |
| G498K | -0.05 | 6.057 | Destabilizing |
| G498R | -0.325 | 6.057 | Destabilizing |
| S499G | 0.2 | 7.998 | Stabilizing |
| S499A | 0.191 | 7.998 | Stabilizing |
| S499T | 0.1 | 7.998 | Stabilizing |
| S499C | -0.183 | 7.998 | Destabilizing |
| S499V | 0.149 | 7.998 | Stabilizing |
| S499L | 0.136 | 7.998 | Stabilizing |
| S499I | 0.125 | 7.998 | Stabilizing |
| S499M | -0.099 | 7.998 | Destabilizing |
| S499P | 0.154 | 7.998 | Stabilizing |
| S499F | 0.017 | 7.998 | Stabilizing |
| S499Y | 0.028 | 7.998 | Stabilizing |
| S499W | -0.172 | 7.998 | Destabilizing |
| S499D | -0.357 | 7.998 | Destabilizing |
| S499E | -0.374 | 7.998 | Destabilizing |
| S499N | 0.027 | 7.998 | Stabilizing |
| S499Q | 0.04 | 7.998 | Stabilizing |
| S499H | -0.084 | 7.998 | Destabilizing |
| S499K | -0.011 | 7.998 | Destabilizing |
| S499R | -0.2 | 7.998 | Destabilizing |
| L606G | 0.13 | 8.002 | Stabilizing |
| L606A | 0.224 | 8.002 | Stabilizing |
| L606T | -0.082 | 8.002 | Destabilizing |
| L606S | -0.072 | 8.002 | Destabilizing |
| L606C | -0.329 | 8.002 | Destabilizing |
| L606V | 0.248 | 8.002 | Stabilizing |
| L606I | 0.247 | 8.002 | Stabilizing |
| L606M | -0.101 | 8.002 | Destabilizing |
| L606P | 0.226 | 8.002 | Stabilizing |
| L606F | -0.032 | 8.002 | Destabilizing |
| L606Y | -0.082 | 8.002 | Destabilizing |
| L606W | -0.283 | 8.002 | Destabilizing |
| L606D | -0.483 | 8.002 | Destabilizing |
| L606E | -0.492 | 8.002 | Destabilizing |
| L606N | -0.195 | 8.002 | Destabilizing |
| L606Q | -0.149 | 8.002 | Destabilizing |
| L606H | -0.279 | 8.002 | Destabilizing |
| L606K | -0.233 | 8.002 | Destabilizing |
| L606R | -0.497 | 8.002 | Destabilizing |
| M607G | -0.098 | 9.473 | Destabilizing |
| M607A | -0.065 | 9.473 | Destabilizing |
| M607T | -0.423 | 9.473 | Destabilizing |
| M607S | -0.407 | 9.473 | Destabilizing |
| M607C | -0.368 | 9.473 | Destabilizing |
| M607V | -0.072 | 9.473 | Destabilizing |
| M607L | -0.082 | 9.473 | Destabilizing |
| M607I | -0.084 | 9.473 | Destabilizing |
| M607P | -0.07 | 9.473 | Destabilizing |
| M607F | -0.237 | 9.473 | Destabilizing |
| M607Y | -0.283 | 9.473 | Destabilizing |
| M607W | -0.402 | 9.473 | Destabilizing |
| M607D | -0.606 | 9.473 | Destabilizing |
| M607E | -0.609 | 9.473 | Destabilizing |
| M607N | -0.49 | 9.473 | Destabilizing |
| M607Q | -0.457 | 9.473 | Destabilizing |
| M607H | -0.483 | 9.473 | Destabilizing |
| M607K | -0.542 | 9.473 | Destabilizing |
| M607R | -0.72 | 9.473 | Destabilizing |
| A609G | 0.124 | 8.678 | Stabilizing |
| A609T | -0.142 | 8.678 | Destabilizing |
| A609S | -0.125 | 8.678 | Destabilizing |
| A609C | -0.35 | 8.678 | Destabilizing |
| A609V | 0.101 | 8.678 | Stabilizing |
| A609L | 0.071 | 8.678 | Stabilizing |
| A609I | 0.07 | 8.678 | Stabilizing |
| A609M | -0.162 | 8.678 | Destabilizing |
| A609P | 0.088 | 8.678 | Stabilizing |
| A609F | -0.121 | 8.678 | Destabilizing |
| A609Y | -0.143 | 8.678 | Destabilizing |
| A609W | -0.332 | 8.678 | Destabilizing |
| A609D | -0.47 | 8.678 | Destabilizing |
| A609E | -0.483 | 8.678 | Destabilizing |
| A609N | -0.217 | 8.678 | Destabilizing |
| A609Q | -0.2 | 8.678 | Destabilizing |
| A609H | -0.234 | 8.678 | Destabilizing |
| A609K | -0.232 | 8.678 | Destabilizing |
| A609R | -0.393 | 8.678 | Destabilizing |
| N610G | 0.404 | 4.35 | Stabilizing |
| N610A | 0.47 | 4.35 | Stabilizing |
| N610T | 0.439 | 4.35 | Stabilizing |
| N610S | 0.44 | 4.35 | Stabilizing |
| N610C | 0.051 | 4.35 | Stabilizing |
| N610V | 0.427 | 4.35 | Stabilizing |
| N610L | 0.401 | 4.35 | Stabilizing |
| N610I | 0.376 | 4.35 | Stabilizing |
| N610M | 0.077 | 4.35 | Stabilizing |
| N610P | 0.437 | 4.35 | Stabilizing |
| N610F | 0.319 | 4.35 | Stabilizing |
| N610Y | 0.357 | 4.35 | Stabilizing |
| N610W | 0.147 | 4.35 | Stabilizing |
| N610D | 0.04 | 4.35 | Stabilizing |
| N610E | 0.061 | 4.35 | Stabilizing |
| N610Q | 0.518 | 4.35 | Stabilizing |
| N610H | 0.207 | 4.35 | Stabilizing |
| N610K | 0.309 | 4.35 | Stabilizing |
| N610R | 0.086 | 4.35 | Stabilizing |
| M611G | -0.024 | 8.437 | Destabilizing |
| M611A | 0.047 | 8.437 | Stabilizing |
| M611T | -0.188 | 8.437 | Destabilizing |
| M611S | -0.188 | 8.437 | Destabilizing |
| M611C | -0.091 | 8.437 | Destabilizing |
| M611V | 0.046 | 8.437 | Stabilizing |
| M611L | 0.037 | 8.437 | Stabilizing |
| M611I | 0.037 | 8.437 | Stabilizing |
| M611P | 0.057 | 8.437 | Stabilizing |
| M611F | -0.126 | 8.437 | Destabilizing |
| M611Y | -0.154 | 8.437 | Destabilizing |
| M611W | -0.292 | 8.437 | Destabilizing |
| M611D | -0.45 | 8.437 | Destabilizing |
| M611E | -0.47 | 8.437 | Destabilizing |
| M611N | -0.27 | 8.437 | Destabilizing |
| M611Q | -0.243 | 8.437 | Destabilizing |
| M611H | -0.262 | 8.437 | Destabilizing |
| M611K | -0.254 | 8.437 | Destabilizing |
| M611R | -0.411 | 8.437 | Destabilizing |
| R613G | 0.043 | 3.618 | Stabilizing |
| R613A | 0.132 | 3.618 | Stabilizing |
| R613T | 0.233 | 3.618 | Stabilizing |
| R613S | 0.118 | 3.618 | Stabilizing |
| R613C | -0.073 | 3.618 | Destabilizing |
| R613V | 0.243 | 3.618 | Stabilizing |
| R613L | 0.258 | 3.618 | Stabilizing |
| R613I | 0.258 | 3.618 | Stabilizing |
| R613M | 0.015 | 3.618 | Stabilizing |
| R613P | 0.201 | 3.618 | Stabilizing |
| R613F | 0.134 | 3.618 | Stabilizing |
| R613Y | 0.252 | 3.618 | Stabilizing |
| R613W | 0.082 | 3.618 | Stabilizing |
| R613D | -0.188 | 3.618 | Destabilizing |
| R613E | -0.165 | 3.618 | Destabilizing |
| R613N | 0.195 | 3.618 | Stabilizing |
| R613Q | 0.187 | 3.618 | Stabilizing |
| R613H | 0.588 | 3.618 | Stabilizing |
| R613K | 0.388 | 3.618 | Stabilizing |
| Q614G | 0.409 | 6.804 | Stabilizing |
| Q614A | 0.411 | 6.804 | Stabilizing |
| Q614T | 0.438 | 6.804 | Stabilizing |
| Q614S | 0.445 | 6.804 | Stabilizing |
| Q614C | 0.033 | 6.804 | Stabilizing |
| Q614V | 0.319 | 6.804 | Stabilizing |
| Q614L | 0.239 | 6.804 | Stabilizing |
| Q614I | 0.214 | 6.804 | Stabilizing |
| Q614M | -0.028 | 6.804 | Destabilizing |
| Q614P | 0.326 | 6.804 | Stabilizing |
| Q614F | 0.193 | 6.804 | Stabilizing |
| Q614Y | 0.194 | 6.804 | Stabilizing |
| Q614W | 0.021 | 6.804 | Stabilizing |
| Q614D | 0.088 | 6.804 | Stabilizing |
| Q614E | 0.093 | 6.804 | Stabilizing |
| Q614N | 0.495 | 6.804 | Stabilizing |
| Q614H | 0.11 | 6.804 | Stabilizing |
| Q614K | 0.322 | 6.804 | Stabilizing |
| Q614R | 0.078 | 6.804 | Stabilizing |
| N679G | -0.433 | 6.851 | Destabilizing |
| N679A | -0.414 | 6.851 | Destabilizing |
| N679T | -0.508 | 6.851 | Destabilizing |
| N679S | -0.504 | 6.851 | Destabilizing |
| N679C | -0.72 | 6.851 | Destabilizing |
| N679V | -0.396 | 6.851 | Destabilizing |
| N679L | -0.392 | 6.851 | Destabilizing |
| N679I | -0.388 | 6.851 | Destabilizing |
| N679M | -0.583 | 6.851 | Destabilizing |
| N679P | -0.381 | 6.851 | Destabilizing |
| N679F | -0.457 | 6.851 | Destabilizing |
| N679Y | -0.427 | 6.851 | Destabilizing |
| N679W | -0.494 | 6.851 | Destabilizing |
| N679D | -0.586 | 6.851 | Destabilizing |
| N679E | -0.577 | 6.851 | Destabilizing |
| N679Q | -0.425 | 6.851 | Destabilizing |
| N679H | -0.544 | 6.851 | Destabilizing |
| N679K | -0.601 | 6.851 | Destabilizing |
| N679R | -0.744 | 6.851 | Destabilizing |
| H680G | -0.309 | 3.704 | Destabilizing |
| H680A | -0.278 | 3.704 | Destabilizing |
| H680T | -0.509 | 3.704 | Destabilizing |
| H680S | -0.502 | 3.704 | Destabilizing |
| H680C | -0.651 | 3.704 | Destabilizing |
| H680V | -0.258 | 3.704 | Destabilizing |
| H680I | -0.256 | 3.704 | Destabilizing |
| H680M | -0.476 | 3.704 | Destabilizing |
| H680P | -0.258 | 3.704 | Destabilizing |
| H680F | -0.326 | 3.704 | Destabilizing |
| H680Y | -0.321 | 3.704 | Destabilizing |
| H680W | -0.425 | 3.704 | Destabilizing |
| H680D | -0.628 | 3.704 | Destabilizing |
| H680E | -0.626 | 3.704 | Destabilizing |
| H680N | -0.517 | 3.704 | Destabilizing |
| H680Q | -0.513 | 3.704 | Destabilizing |
| H680K | -0.544 | 3.704 | Destabilizing |
| H680R | -0.673 | 3.704 | Destabilizing |
| W726G | -0.266 | 19.244 | Destabilizing |
| W726A | -0.246 | 19.244 | Destabilizing |
| W726T | -0.178 | 19.244 | Destabilizing |
| W726S | -0.19 | 19.244 | Destabilizing |
| W726C | -0.381 | 19.244 | Destabilizing |
| W726V | -0.21 | 19.244 | Destabilizing |
| W726L | -0.241 | 19.244 | Destabilizing |
| W726I | -0.202 | 19.244 | Destabilizing |
| W726M | -0.413 | 19.244 | Destabilizing |
| W726P | -0.227 | 19.244 | Destabilizing |
| W726F | -0.223 | 19.244 | Destabilizing |
| W726Y | -0.204 | 19.244 | Destabilizing |
| W726D | -0.439 | 19.244 | Destabilizing |
| W726E | -0.436 | 19.244 | Destabilizing |
| W726N | -0.179 | 19.244 | Destabilizing |
| W726Q | -0.179 | 19.244 | Destabilizing |
| W726H | -0.232 | 19.244 | Destabilizing |
| W726K | -0.15 | 19.244 | Destabilizing |
| W726R | -0.185 | 19.244 | Destabilizing |
| H1034G | -0.081 | 9.801 | Destabilizing |
| H1034A | -0.069 | 9.801 | Destabilizing |
| H1034T | -0.274 | 9.801 | Destabilizing |
| H1034S | -0.257 | 9.801 | Destabilizing |
| H1034C | -0.465 | 9.801 | Destabilizing |
| H1034V | -0.086 | 9.801 | Destabilizing |
| H1034L | -0.109 | 9.801 | Destabilizing |
| H1034I | -0.11 | 9.801 | Destabilizing |
| H1034M | -0.292 | 9.801 | Destabilizing |
| H1034P | -0.098 | 9.801 | Destabilizing |
| H1034F | -0.212 | 9.801 | Destabilizing |
| H1034Y | -0.206 | 9.801 | Destabilizing |
| H1034W | -0.309 | 9.801 | Destabilizing |
| H1034D | -0.542 | 9.801 | Destabilizing |
| H1034E | -0.548 | 9.801 | Destabilizing |
| H1034N | -0.242 | 9.801 | Destabilizing |
| H1034Q | -0.291 | 9.801 | Destabilizing |
| H1034K | -0.243 | 9.801 | Destabilizing |
| H1034R | -0.181 | 9.801 | Destabilizing |
| H1035G | 0.033 | 8.828 | Stabilizing |
| H1035A | 0.066 | 8.828 | Stabilizing |
| H1035T | -0.201 | 8.828 | Destabilizing |
| H1035S | -0.189 | 8.828 | Destabilizing |
| H1035C | -0.403 | 8.828 | Destabilizing |
| H1035V | 0.097 | 8.828 | Stabilizing |
| H1035L | 0.078 | 8.828 | Stabilizing |
| H1035I | 0.078 | 8.828 | Stabilizing |
| H1035M | -0.155 | 8.828 | Destabilizing |
| H1035P | 0.075 | 8.828 | Stabilizing |
| H1035F | 0.014 | 8.828 | Stabilizing |
| H1035Y | 0.005 | 8.828 | Stabilizing |
| H1035W | -0.106 | 8.828 | Destabilizing |
| H1035D | -0.5 | 8.828 | Destabilizing |
| H1035E | -0.501 | 8.828 | Destabilizing |
| H1035N | -0.2 | 8.828 | Destabilizing |
| H1035Q | -0.222 | 8.828 | Destabilizing |
| H1035K | -0.158 | 8.828 | Destabilizing |
| H1035R | -0.118 | 8.828 | Destabilizing |
| K1040G | 0.216 | 9.562 | Stabilizing |
| K1040A | 0.268 | 9.562 | Stabilizing |
| K1040T | -0.006 | 9.562 | Destabilizing |
| K1040S | -0.015 | 9.562 | Destabilizing |
| K1040C | -0.282 | 9.562 | Destabilizing |
| K1040V | 0.288 | 9.562 | Stabilizing |
| K1040L | 0.236 | 9.562 | Stabilizing |
| K1040I | 0.243 | 9.562 | Stabilizing |
| K1040M | -0.041 | 9.562 | Destabilizing |
| K1040P | 0.284 | 9.562 | Stabilizing |
| K1040F | 0.153 | 9.562 | Stabilizing |
| K1040Y | 0.132 | 9.562 | Stabilizing |
| K1040W | -0.03 | 9.562 | Destabilizing |
| K1040D | -0.393 | 9.562 | Destabilizing |
| K1040E | -0.383 | 9.562 | Destabilizing |
| K1040N | -0.058 | 9.562 | Destabilizing |
| K1040Q | -0.013 | 9.562 | Destabilizing |
| K1040R | -0.287 | 9.562 | Destabilizing |
